# Plant Bioelectronics and Biohybrids: The Growing Contribution of Organic Electronic and Carbon-Based Materials

**DOI:** 10.1021/acs.chemrev.1c00525

**Published:** 2021-12-20

**Authors:** Gwennaël Dufil, Iwona Bernacka-Wojcik, Adam Armada-Moreira, Eleni Stavrinidou

**Affiliations:** † Laboratory of Organic Electronics, Department of Science and Technology, Linköping University, SE-601 74 Norrköping, Sweden; ‡ Wallenberg Wood Science Center, Department of Science and Technology, Linköping University, SE-60174 Norrköping, Sweden; § Umeå Plant Science Centre, Department of Forest Genetics and Plant Physiology, Swedish University of Agricultural Sciences, Campus Umeå, SE-901 83 Umeå, Sweden

## Abstract

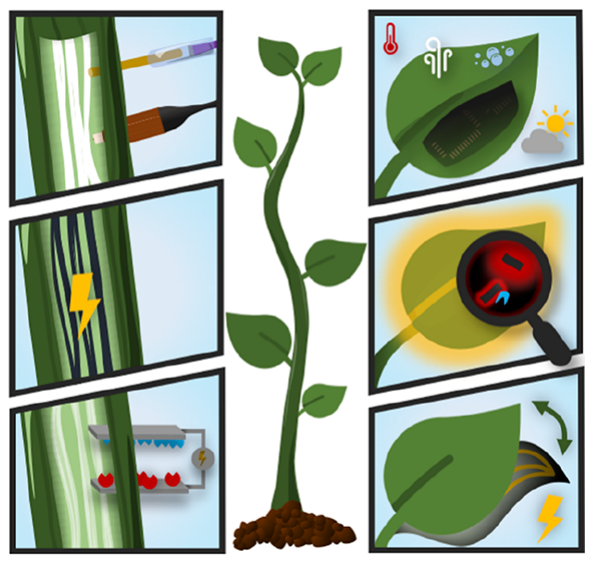

Life in our planet is highly dependent on plants as they are the primary source of food, regulators of the atmosphere, and providers of a variety of materials. In this work, we review the progress on bioelectronic devices for plants and biohybrid systems based on plants, therefore discussing advancements that view plants either from a biological or a technological perspective, respectively. We give an overview on wearable and implantable bioelectronic devices for monitoring and modulating plant physiology that can be used as tools in basic plant science or find application in agriculture. Furthermore, we discuss plant-wearable devices for monitoring a plant’s microenvironment that will enable optimization of growth conditions. The review then covers plant biohybrid systems where plants are an integral part of devices or are converted to devices upon functionalization with smart materials, including self-organized electronics, plant nanobionics, and energy applications. The review focuses on advancements based on organic electronic and carbon-based materials and discusses opportunities, challenges, as well as future steps.

## 1 Introduction

Plants are an indispensable part of our ecosystem and are vital for our survival. Through photosynthesis, plants convert sunlight to chemical energy and regulate the concentration of carbon dioxide and oxygen in the atmosphere, thus creating favorable conditions that support life on our planet. Plants also play an important role in human development, not only being the primary source of food but also providing humanity with a wealth of materials such as fibers for clothing, wood for fuel, construction, and paper, and other high-value molecules for medicines and cosmetics. Moreover, plants are essential for our well-being, in nature or integrated within the urban environment, offering peace of mind, spectacular views and connection to our natural habitat.

Over the last decades, plants have been suffering due to the climate crisis that results in temperature rise, droughts, floods, and sea level rise. These harsh environmental conditions result in a low yield of agricultural production and desertification.^1^ A recent report of the FAO (Food and Agriculture Organization) on food security indicates that the number of people that are affected by hunger has been increasing since 2014.^2^ Malnutrition is still a major challenge for millions of people, and the goal for zero hunger by 2030 will most probably not be achieved. Although this is a result of a complex socioeconomic framework, the effect of climate change in agriculture is an important factor. Furthermore, mortality of trees is rising caused by both direct and indirect effects of climate change such as fires, pests, and pathogens.^3^ Forests capture 2.4 Pg carbon per year, about 25% of anthropogenic emissions,^4^ but also serve as a unique source of materials of high economic value and have been suggested as one of the most effective strategies to address the climate change.^5^ Without a doubt, attention must be given to increasing plants productivity and nutritional content and understanding how plants respond and acclimate to abiotic and biotic stress.

Basic plant biology research relies on sophisticated molecular and genetic in vivo methods but also on highly invasive tissue sampling followed by ex vivo analysis. One of the main drawbacks of genetic engineering is that it is mostly developed in model species, and extension to other species is not always straightforward. Invasive sampling, on the other hand, disturbs plant signaling, and consequently, many ex vivo analyzed samples do not correspond to the natural status of the plant. Bioelectronic technologies can complement conventional methods and offer new possibilities for real-time monitoring and dynamic modulation of plant physiology. Bioelectronic sensors can translate complex biological inputs to electronic readout signals, while bioelectronic actuators can modulate biological networks via electronic addressing.^6,7^ In particular, devices based on organic electronic materials as active layers offer advantages of signal transduction due to their mixed ionic/electronic conduction that enables an intimate communication with the inherently ionic biological milieu.^8,9^ However, the field of bioelectronics is mainly driven by biomedicine for developing new therapies and diagnostics tools. Therefore, these technologies have not been applied to the same extent in plants as in animals. Several reasons may have contributed to this, such as no historic/traditional background, the focus of plant biology science on genetic methods, less research funding in plant science, and less interaction between plant biology and electronic and engineering disciplines.

Bioelectronics can also find application in smart and precision agriculture.^10^ Motivated by increasing food demands and sustainability goals, precision agriculture uses distal or proximal sensors to optimize production by addressing changes of the growth environment via predictive or reactive approaches. Currently, climate sensing has low spatial resolution, while very limited information can be obtained for plant physiology. Bioelectronic technologies can be used to monitor the microenvironment of plants in both air and soil, giving information on high spatial resolution, even reaching the single plant level. Furthermore, sensors and actuators can be integrated in plants for monitoring and modulating vital parameters related to growth, product quality, and stress responses, enabling the farmer to make informed decisions. Bioelectronic devices based on organic or carbon materials can be fabricated with low-cost mass production approaches such as screen printing that is advantageous for large-scale applications.^11−14^

Another relatively new area of research is plant biohybrids, where plants are viewed from a technological perspective. Plants are amazing machines powered by the sun that can self-repair, sense, and adapt to their environment and have hierarchical structures and complex biochemistry. Furthermore, plants are very resilient to a wide range of modifications; biological chimera (hybrid) plants have been formed since ancient times via grafting. Plants have been proven resilient to modifications with organic electronic and carbon materials processed from aqueous solutions. The functional properties of these materials in combination with their self-organization or spontaneous localization in various plant tissues enabled the development of biohybrids systems with device operation for example in energy and sensing applications.

**Figure 1:**
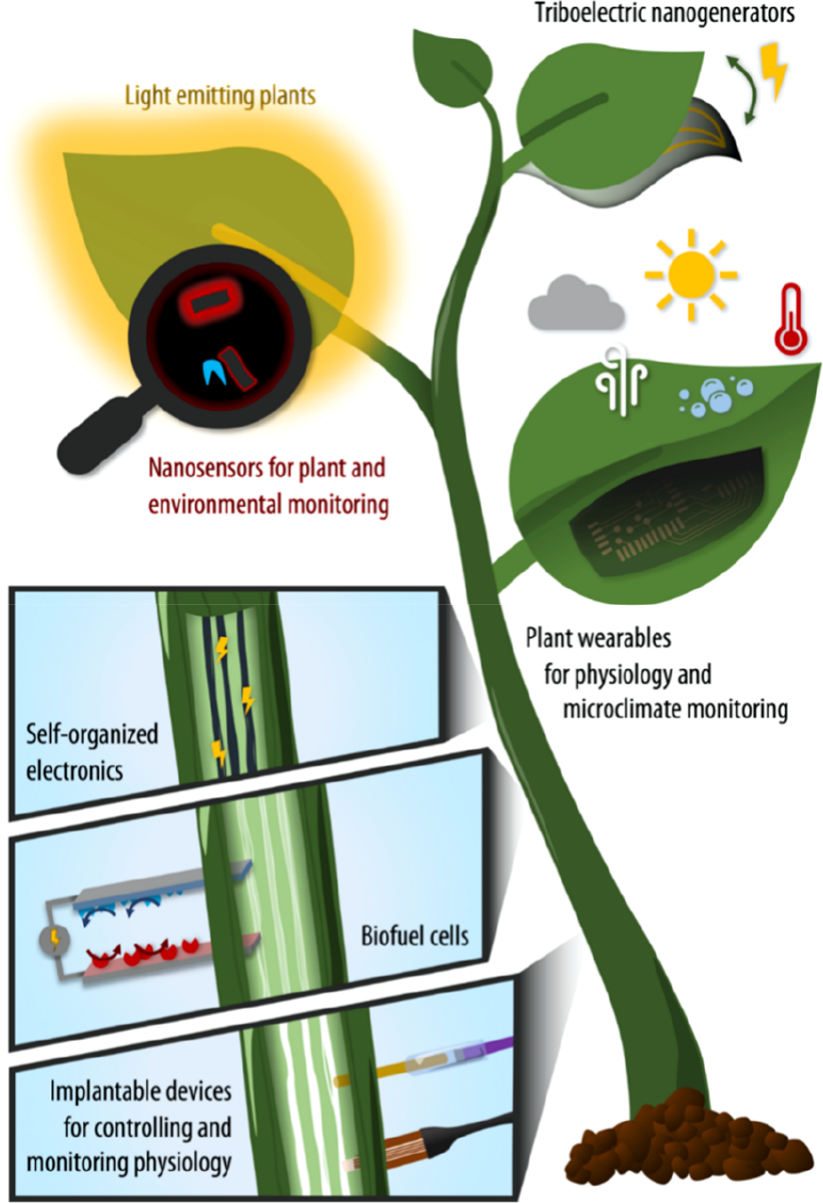
Plant bioelectronic and biohybrid applications overview.

In this work, we will review bioelectronic technologies for plants and biohybrid systems based on plants (Figure 1). The review has a pedagogical character aiming to introduce these technologies to scientists of a variety of disciplines. We overview wearable and implantable bioelectronic devices for plant physiology and discuss them within the context of basic plant science and/or agricultural applications. Wearable devices for monitoring a plant’s microclimate will also be presented. Furthermore, we will cover developments in the area of plant biohybrid systems with examples on self-organized electronics, plant nanobionics, and energy harvesting. The review mainly includes works based on organic electronic and carbon materials aiming to highlight the contribution of these materials in the field. Works based on other type of materials such as inorganic conductors or organic dielectrics are occasionally included for comparison or if they dominate an important area of application.

In addition, we will introduce basic concepts on plant biology and discuss in more detail electrical signaling in plants. Finally, we will present the main challenges of the field and give future perspectives.

### 1.1 Basics of Plant Biology

**Figure 2:**
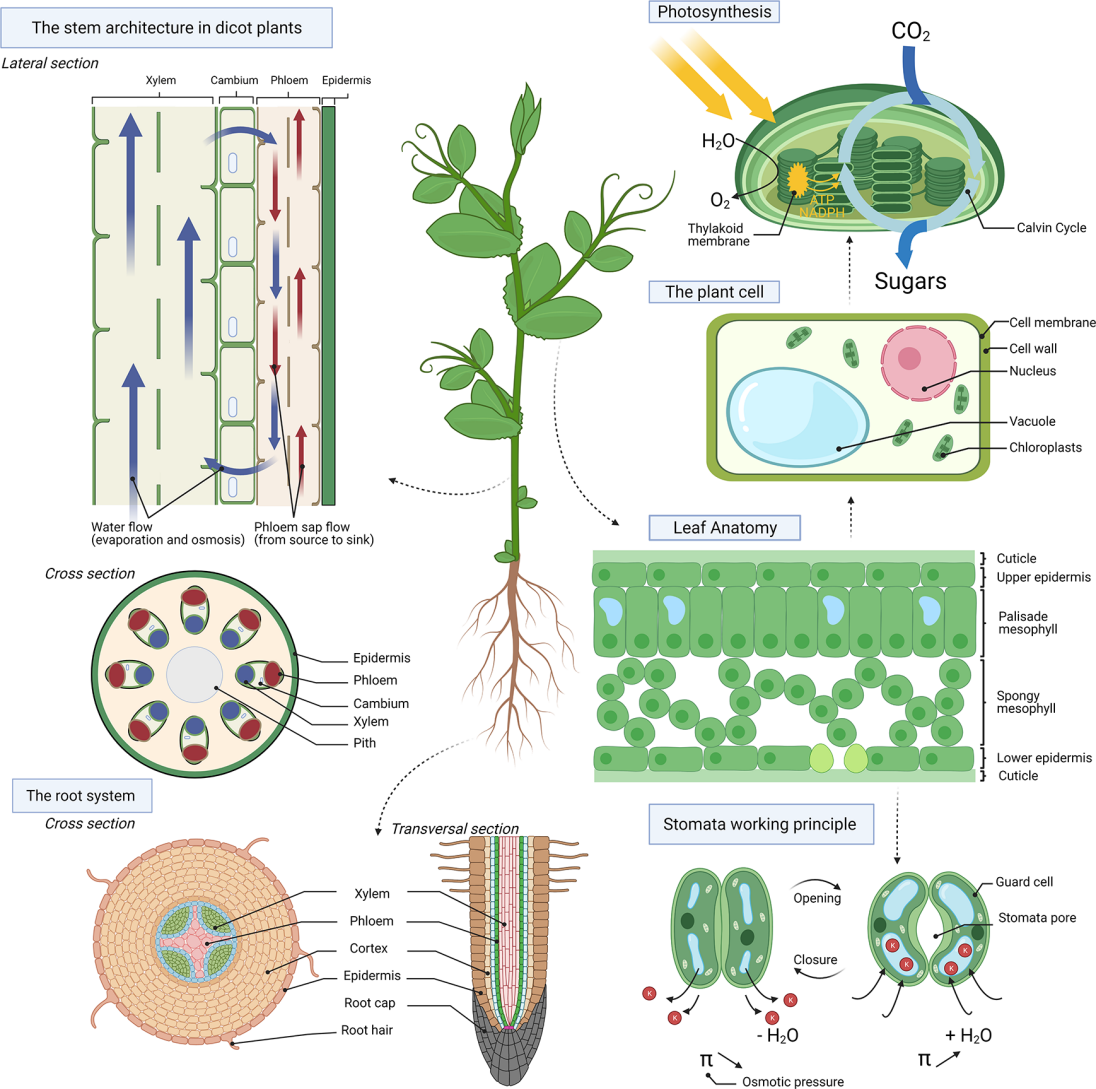
Plant anatomy.

In this section, we give a very brief introduction to plant anatomy (Figure 2), refreshing the readers’ memory of basic plant functions and introducing terminology that will aid the discussion of the various technologies and applications in the next sections.

The anatomy of the plant can be understood in relation to the evolutionary process with the transition from sea to land as it had to evolve to survive in land.^15^ The roots are responsible for uptake of water and minerals from the soil while at the same time anchor the plant to the ground. The stem carries the main photosynthetic organs, the leaves, extending them toward the light source while it connects roots and leaves. The vascular system forms a microfluidic network that is distributed throughout the plant and consists of the xylem and phloem tissue. The xylem is based on dead cells and is mostly responsible for the transport of water from roots to shoot. When the water reaches the leaves, it enters the apoplastic space, a porous area in the leaves, and then evaporates through the stomata, the pores on the leaf surface, in a process called transpiration. Stomata consist of two specialized cells, the guard cells that open and close the pore aperture by changing their turgor pressure. Stomata function is regulated by physical and biochemical stimuli as the plant tries to optimize their opening in order to minimize water loss but at the same time be able to exchange gases for photosynthesis and respiration.

Photosynthesis is a major process that defines the evolution of the planet and powers directly or indirectly most forms of life. “What drives life is. . . a little current, kept up by the sunshine” were the words of Albert Szent-Györgyi, Nobel Prize in Physiology or Medicine in 1937, capturing the importance of photosynthesis.^15^ Photosynthesis takes place at chloroplasts with light-dependent reactions occurring at the thylakoids that are lipid membranes with embedded photosynthetic proteins. Along the thylakoids a series of electrochemical reactions take place all powered by the sunlight. Starting from the photoexcitation of photosystem II, the light-dependent reactions result in the synthesis of ATP and the reduction of the coenzyme NADP^+^ to NADPH. The energy of ATP and the reducing power of NADPH are then used for the synthesis of sugars from CO_2_ in the Calvin cycle (light-independent reactions). The sugars produced by photosynthesis are then distributed to the plant via the phloem vascular tissue to cover the energy demands, growth, and development, while any excess is stored in the form of starch. Therefore, the phloem can distribute sugars from source to sink tissues.

Another unique feature of plant cells is their cell wall that ensures mechanical stability to the plant and ability to sustain major water loss without dying. The plant cell walls have a multilevel hierarchical structure that consists of crystalline cellulose fibers embedded in amorphous matrix of hemicellulose and lignin. Cell wall components are the most abundant biopolymers on earth, representing high value materials. The density of cell walls can be tuned dynamically, loosening for cell elongation and densifying for protection from elicitors.

Plants grow throughout their lives; growth is initiated in meristems that consist of stem cells able to differentiate into the various cells. Apical meristems are based on the tip of the shoots and root. In this way, the roots can reach into new soil territories to find water while the photosynthetic parts can grow to reach more effectively the light. Lateral meristems, vascular and cork cambium, on the other hand, are responsible for the thickening of the stem and shoot.

Signaling in plants mainly occurs via biomolecules that are either synthesized locally in the tissue of action or in distant tissues. One of the major classes of plant-signaling molecules are phytohormones that are present in low concentrations and regulate plant growth and development in many different levels acting synergistically or antagonistically. Phytohormones are also important regulators of plant responses and acclimation to abiotic and biotic stress. Other molecules involved in signaling include peptides and mRNAs. Over the past decade, there have been significant advancements in identifying fast long-distance signaling in plants as well. Fast signaling is mediated by Ca^2+^ waves, reactive oxygen species (ROS) waves, and electrical signals. In the Plants and Electricity section, we give a brief overview of the current understanding of electrical signaling in plants.

## 2 Bioelectronic Devices for Monitoring the Plant Microenvironment

Plants, being sessile organisms, must coordinate their growth and development depending on changes in their environment. Continuous monitoring of plants’ local environment and correlation with their physiological status can enable better understanding of plants’ acclimation and adaptability. This becomes particularly important nowadays where climate change is affecting crops and forests. In order to provide food security for a growing population and maintain healthy forests for carbon sequestration in the upcoming decades, a better understanding of how plants respond to various abiotic and biotic stresses is needed. In this way, not only growth conditions but also breeding and genetic engineering can be optimized. These aspects can lead to plants with an increased stress tolerance and an enhanced productivity even in suboptimal growth conditions.

In agriculture, the climate is usually monitored by centralized sensors for temperature and humidity. Smart or precision agriculture approaches, though, are receiving more and more attention. The aim is to increase the production yield in a sustainable manner^16^ by remotely tracking the plants’ microenvironment and by integrated decision-making process for optimizing the rational distribution of limited resources. For example, hyperspectral drones can be used to remotely detect the early stages of a disease and give information on plants’ water status. High-tech closed greenhouses and vertical indoor farms have started to emerge, enabling integration of multiple climate sensors into feedback loop systems for optimizing the growth conditions. One of the challenges of smart agriculture is to collect and process a large amount of data and translate them into specific actions that will guide farm management. AI start-ups are developing software for analyzing multiple sensors readings and translating them to decisions for the farmer.^17^ The widespread distribution of lightweight microclimate sensory devices at the single-plant level can help to determine optimal growth conditions for each individual plant not only in agriculture but also in research, for example, to correlate plant phenotype with growth parameters in high-throughput phenotyping facilities.

**Figure 3:**
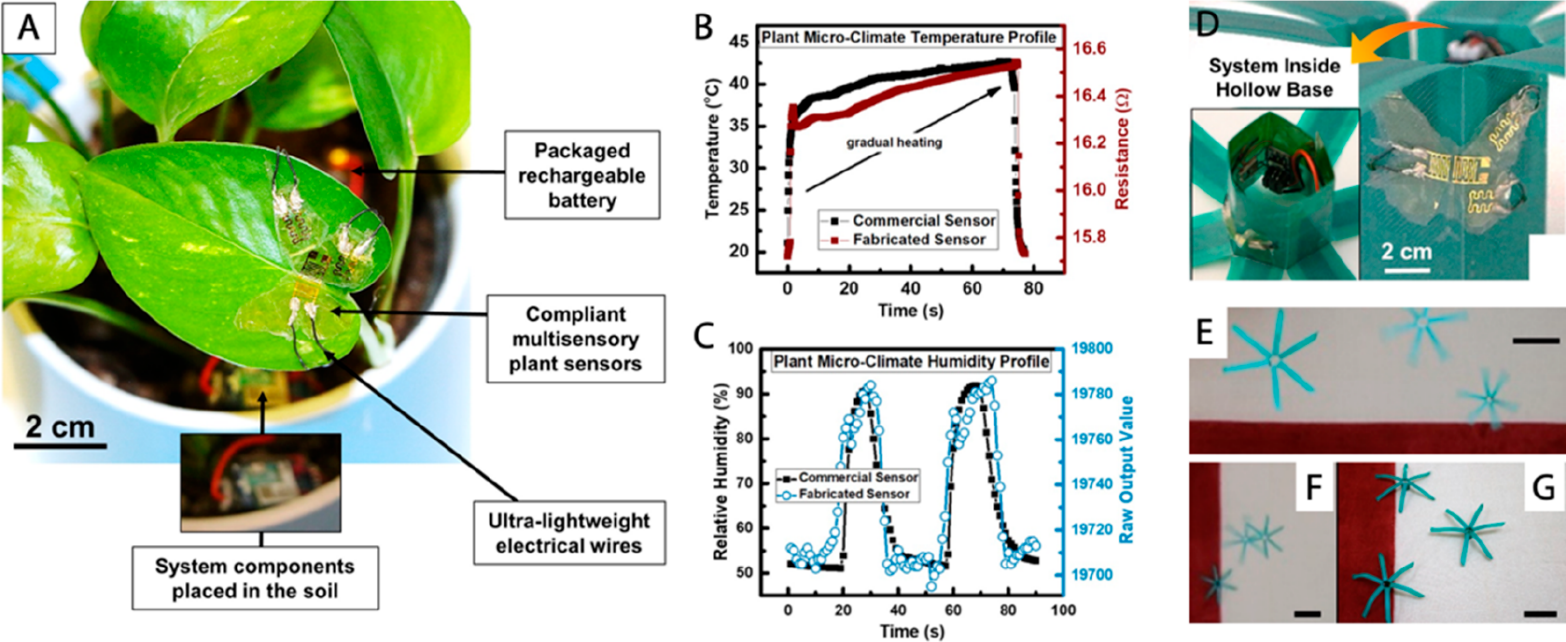
Flexible plant wearable system for real time remote sensing of ambient temperature and humidity. (A) Photograph of the sensors patterned on butterfly shape PDMS substrate attached to the leaf surface by conformable adhesion. The device was integrated with battery using ultra lightweight cables forming an autonomous portable platform. (B,C) Performance of developed plant wearable Au-based temperature sensor and polyimide-based RH sensors with comparison to the commercial counterparts. (D) “PlantCopter”, a 3D printer origami assembled structure for efficient distribution of the ambient monitoring platform along the field, mimicking the flight of dandelion seeds. Butterfly device and system components were attached to the base of the copter acting as the center of mass. (E–G) Images of three “Plant Copters” released in free-fall motion depicting their smooth landing. Scale bar: 10 cm. Adapted with permission from ref (16). Copyright 2018 Springer US under CC BY 4.0 (http://creativecommons.org/licenses/by/4.0/).

Below, an overview of the current state of the art on microclimate monitoring is presented, focusing only on plant-wearable devices while advances on sensors that are distributed in the field are beyond the scope of this review. The reader interested in this topic may refer to other previous works.^18,19^ To enable long-term attachment to uneven, constantly growing plant tissues, plant-wearable sensors should be flexible, preferably stretchable and lightweight to reduce the impact on plant physiology. The first plant-wearable system for continuous remote sensing of ambient temperature, humidity and plant growth (the latter is discussed in section 3.2, Plant Growth) was developed in 2018 by Nassar et al. (Figure 3A).^16^ The sensors were patterned on a polyimide layer on 50 μm thick butterfly-shaped polydimethylsiloxane (PDMS) substrate that attaches conformally to the irregular leaf surface. Relative humidity (RH) sensing was based on the humidity-dependent capacitance of polyimide, while temperature sensing was based on a meander-structured Au thermistor. Using ultralight electrical wires, this flexible and stretchable sensing platform was connected to a rechargeable battery and a chip that could save the data in local memory or transmit wirelessly to a smartphone via a low power Bluetooth transceiver. Attached to the leaf surface (plant model not specified), the wearable platform was able to monitor temperature and RH in real time, demonstrating response and recovery time similar to commercial state of the art counterparts (Figure 3B,C), although it is not clear whether the experiments were performed in indoor or outdoor conditions. As, in field conditions, tagging each plant would be very labor intensive and time-consuming, the authors have also engineered an ultralightweight and low-cost copter for distributing these sensors along the field. The “PlantCopter”, an origami-assembled 3D printed structure, was designed to spin and smoothly land in the field, mimicking dandelion seeds that spread with the wind (Figure 3D–G). The butterfly-shaped multisensor was attached on the outer shell base of the PlantCopter while the system components were placed within its hollow base, also acting as the center of mass. Such ambient monitoring copters could be distributed in the field using a drone.

**Figure 4:**
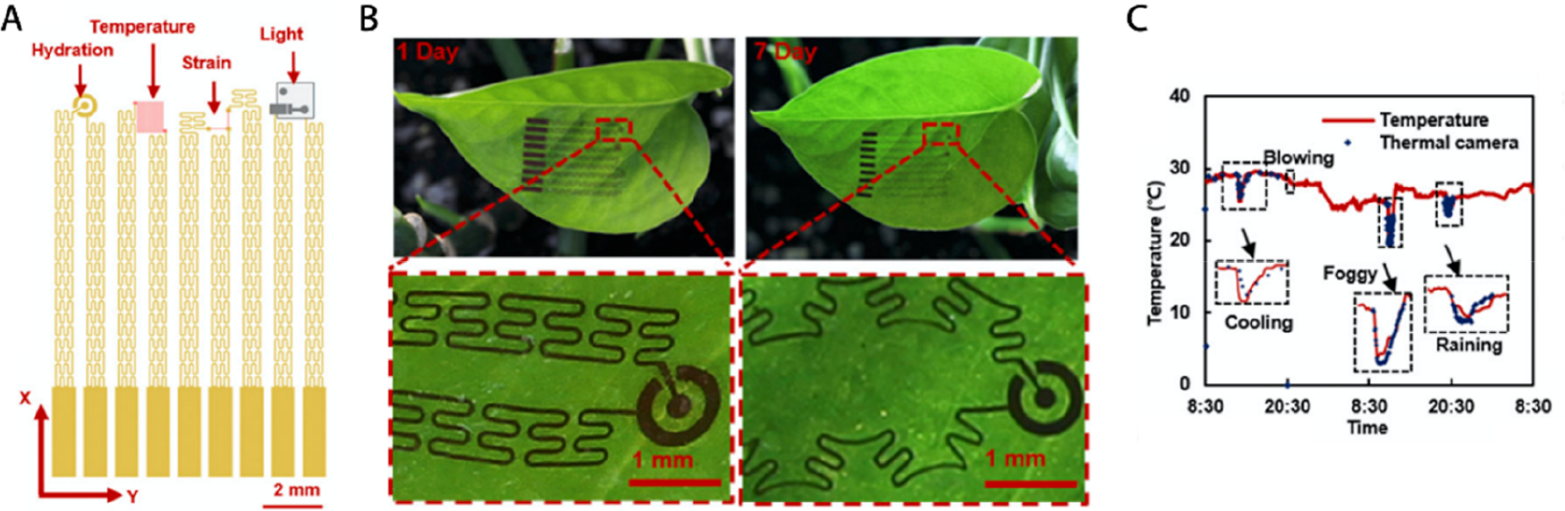
Multiparameter monitoring system that is able to synchronize its growth with the host leaf thanks to the use of stretchable porous silicone substrate and serpentine-shaped interconnectors. (A) Schematics of the sensing elements for monitoring RH (Cu capacitor), temperature (Ti/Cu thermistor), strain (CNTs-based strain gauges), and light illumination (silicon phototransistor). (B) Photographs and optical micrographs of the device attached to *Scindapsus aureus* leaves directly after attachment and after 7 days of implementation. (C) Monitoring of indoor ambient conditions for 2 days using the developed plant wearable platform. Reprinted from ref (20). Copyright 2019 American Chemical Society.

In order to limit failure under physical stresses induced by both plant growth and plant physiology, Zhao et al. engineered a multiparameter stretchable sensor that is able to synchronize its growth with the host leaf (Figure 4).^20^ The stretchability was obtained thanks to the use of a stretchable porous silicone substrate and serpentine-shaped interconnectors. The perforated silicone substrate provided good adhesion to the leaf surface and a high permeability to light, gas, and water vapor, thus minimizing the device’s influence on plant physiology. The platform was able to monitor temperature, RH, strain, and light intensity. A Ti/Cu thermistor with a meander-like structure was used for temperature sensing from 10 to 62 °C, a Cu capacitor for full range RH monitoring, two perpendicular carbon nanotubes (CNTs) strain gauges for strain sensing, and a commercially available silicon phototransistor for the light intensity. Remote data collection and transmission via a wireless sensing circuit with a Zigbee protocol was also demonstrated in this example as it is a vital consideration for single-level plant sensors. The multisensor was evaluated on golden pothos (*Scindapsus aureus*) in a controlled environment where the conditions were changed deliberately while continuous monitoring was demonstrated for 1 week. Ambient monitoring results obtained using the plant-wearable platform are comparable to the response of commercial devices. In a proof-of-concept experiment, the sensor was also applied in outdoor settings on a corn plant and the microclimate was monitored for 2 h.

Another example of a plant-wearable microclimate sensor is the work of Lu et al. that demonstrated a flexible system for real time probing of leaf humidity (i.e., transpiration; discussed in section 3.1, Transpiration) and three environmental parameters: ambient humidity, temperature, and the light irradiation intensity.^21^ The system was based on a 50 μm thick polyimide substrate with a cross shape, where the three arms incorporate sensors and the fourth arm is integrated with a readout system. The arm with a plant transpiration sensor was attached to the leaf lower epidermis using medical tape, while other arms were suspended in air. Interdigital graphene electrodes were formed on the polyimide substrate by laser scanning. ZnIn_2_S_4_ nanosheets were deposited by drop-casting between graphene electrodes to form a humidity-sensing element. ZnIn_2_S_4_ nanosheets with silver electrodes were used also for the optical sensors that were based on the photoconductivity of the semiconductor. The ZnIn_2_S_4_ nanosheet sensors exhibited remarkably fast light response and stable, durable resistive RH sensing from 30 to 90%. Temperature sensing was achieved via SnO_2_ nanoparticles and single-walled carbon nanotubes in a thermistor structure. The system attached to a *Pachira macrocarpa* leaf enabled real-time monitoring of ambient RH, temperature, light, and plant hydration for 16 days under indoor conditions without observable performance degradation.

All three developed systems for plant-wearable ambient monitoring show promising performance in indoor conditions; however, they have been barely applied in outdoor settings. As such, the influence of rain, wind, variable humidity, temperature, and light intensity should be investigated.

To the best of our knowledge, organic electronics have not been applied in plant-wearable microclimate sensors. In the literature, though, there are numerous examples of temperature and humidity sensors based on organic semiconductors as active materials. Conjugated polymers can be processed from solution and thus can be deposited via large-scale fabrication methods such as printing and spray coating that are promising for developing low-cost devices. Furthermore, conjugated polymers are semitransparent and are compatible with lightweight conformable and stretchable substrates, which is beneficial for plant wearables.

For temperature sensing, several organic devices based on conducting polymer electrodes^22−26^ or organic thin film transistors^27,28^ have been demonstrated, including skin-wearable devices. In doped organic semiconductors, when temperature increases the conductivity increases as described by thermally activated hopping conduction.^29^ For example, a flexible and wearable temperature sensor based on poly(3,4-ethylenedioxythiophene):polystyrenesulfonate (PEDOT:PSS) printed on a flexible substrate was able to linearly respond to temperature changes in the range of 30–80 °C with stable performance after bending 300 times.^30^ One of the most sensitive PEDOT:PSS-based temperature sensors, reported so far, was based on PEDOT:PSS film on the PDMS substrate. The fabrication parameters were optimized to generate microcracks.^31^ Interestingly, this sensor showed an increase in resistance with temperature, which was attributed to the thermal expansion of the PDMS substrate that caused the expansion of the cracks in the PEDOT:PSS film. A temperature sensor based on PEDOT:PSS and carbon nanotubes has also been integrated with a touch sensor and a drug-delivery system, forming a “smart bandage”.^25^

PEDOT-based wearable temperature sensors were mostly developed for health monitoring and therefore are often characterized for temperatures above 30 °C, which is only partly relevant for microclimate monitoring applications. Transferring these technologies to plant-wearable climate monitoring will require tuning of the sensor performance for the temperature range between −20 and +50 °C. A recently developed PEDOT-based temperature sensor with a fluoropolymer protective layer enabled sensitive and stable detection of temperature in a range from −15 to +80 °C,^26^ thus within the relevant temperature range for climate monitoring.

Several humidity sensors based on thin-film PEDOT:PSS have also been recently developed based on RH-dependent resistance. With increasing RH, more water molecules will be absorbed on hydrophilic PSS shells, resulting in the increase of the distance between PEDOT chains and consequent decrease of the film conductance. However, at RH above 80%, the resistance decreases with RH, probably due to formation of a water meniscus layer which dissolves PSS protons. To improve RH sensor performance, conjugated polymers are often combined with other materials. For example, PEDOT:PSS combined with graphene oxide showed an outstanding sensitivity with good thermal and mechanical stability for RH range of 25–85%.^32^ On the other hand, the combination of three active materials, PEDOT:PSS, Methyl Red, and graphene oxide, allowed the detection of a full RH range.^33^

An organic electronic multiparameter sensor able to sense temperature, RH, and pressure with minimal crosstalk has been engineered using a single device configuration based on PEDOT:PSS/cellulose aerogels.^34^ The sensors took advantage of the ionic and electronic thermoelectric phenomenon that induced different dynamics of RH and T-induced voltage changes, and therefore, could distinguish between the various inputs.

As organic electronic technologies are compatible with cost-effective fabrication techniques and demonstrate promising performance in the mentioned above sensing applications, they are promising candidates for low-cost plant wearable sensors for monitoring microclimate and plant growth.

## 3 Bioelectronic Devices for Monitoring Plant Physiology

Monitoring plant physiology with a high spatiotemporal resolution will contribute toward a deeper mechanistic understanding of plant biological processes. Plant phenotype is usually evaluated manually by measuring the weight and the dimensions, while imaging combined with specialized software can semiautomate the process. Fully automated platforms for high-throughput phenotyping also exist, where plants are placed on conveyor belts with RFID tags and can be automatically imaged, weighted, watered, and fertilized during their growth. Integration of IR cameras in such platforms can give information on transpiration, while CCD cameras coupled with pulse amplitude modulation (PAM)-fluorometry can determine photosynthetic activity. Usually, these facilities exist in state-of-the-art Plant Science Research Institutes such as the tree phenotyping facility of Umeå Plant Science Center in the north of Sweden where 350 trees up to 2.5 m high can be automatically phenotyped.^35^

Transpiration and photosynthesis usually are measured manually with hand-held gas-exchange instruments and fluorometry instruments, respectively. Metabolites and other biomolecules are commonly analyzed via destructive methods where the tissue of interest is collected from the plant, processed, and then analyzed via chromatography/mass spectrometry. While in biomedicine there is a lot of focus on the development of point of care sensors, these technologies and concepts are barely explored in plant biology. However, bioelectronic sensors for plants have started to emerge with implantable sensors and epidermal/wearable sensors that are attached on leaf or stem. Epidermal devices are minimally invasive but can only give information on parameters that can be accessed via the epidermis of the plant. Implantable devices, on the other hand, can provide tissue-specific information, but currently only a handful of examples exist. In both cases, though, attention should be given to minimize the plant response after sensor integration to not affect the plant physiological status.

### 3.1 Transpiration

Monitoring stomatal conductance, a measure of the degree of stomata opening, enables the determination of plants’ water consumption (transpiration) and gives indications on the plant water status. For example, the stomata close in drought conditions, minimizing transpiration to prevent water loss, but at the same time this results in a reduction of the photosynthetic rate and plant growth. By measuring transpiration and carbon assimilation, the water use efficiency of the plant can be determined. This can then be used as a figure of merit for plants’ productivity with minimal water use. One of the most common methods for measuring stomata aperture is mold impression from a leaf surface (e.g., using nail polish), however this method does not enable kinetic studies and it is destructive for the leaf. Determination of stomata aperture via microscopy in intact plants enables real time monitoring but requires the plant to be mounted under the microscope; therefore, it prevents monitoring it in its natural growth environment. State of the art transpiration analyzers are hand-held gas exchange instruments that can be applied even in field conditions. A chamber is attached on a defined leaf area (up to 36 cm^2^), and the instrument measures water vapor, temperature, and CO_2_ from which transpiration rate and stomatal conductance can be calculated. Moreover, the gas-exchange instruments can be complemented with a fluorometer, enabling measurement of chlorophyll fluorescence, which can be a quantitative marker of plant health. However, in order to collect reliable data, proper calibration of the device has to be done and the chamber environment should mimic the ambient environment.^36^ Furthermore, these instruments are expensive and bulky, and analysis of multiple plants is challenging. All these limitations impose the need to develop methods and tools for measuring stomata function in real time, over long periods, and in their natural growth environment.

Koman et al. have developed a resistive sensor that enables real-time monitoring of stomata aperture with a single stoma precision.^36^ The device consists of conductive lines patterned directly on the leaf connected to conductive micropillars mounted on the guard cells of the stomata. When stomata are open, the distance between guard cells increases, resulting in the loss of the electrical contact between the two conducting micropillars, and consequently, in a high resistance output. Inversely, when stomata close, an electrical contact is established, and the resistance decreases significantly. The electrodes were based on an aqueous carbon nanotube ink and were patterned directly on the leaves with the use of a microfluidic chamber. The sensor was able to track stomata aperture changes over 1 week detecting their diurnal changes as well as determining opening/closure speed under both normal and drought conditions. The unprecedented electronic single stoma resolution provides great insight on stomata responses that were not observable with conventional methods such as water vapor monitoring and the latency on the stomatal response to light and day-night cycle. However, the direct printing on the leaf and the manual positioning of the conducting micropillars make this approach applicable only for research purposes.

**Figure 5:**
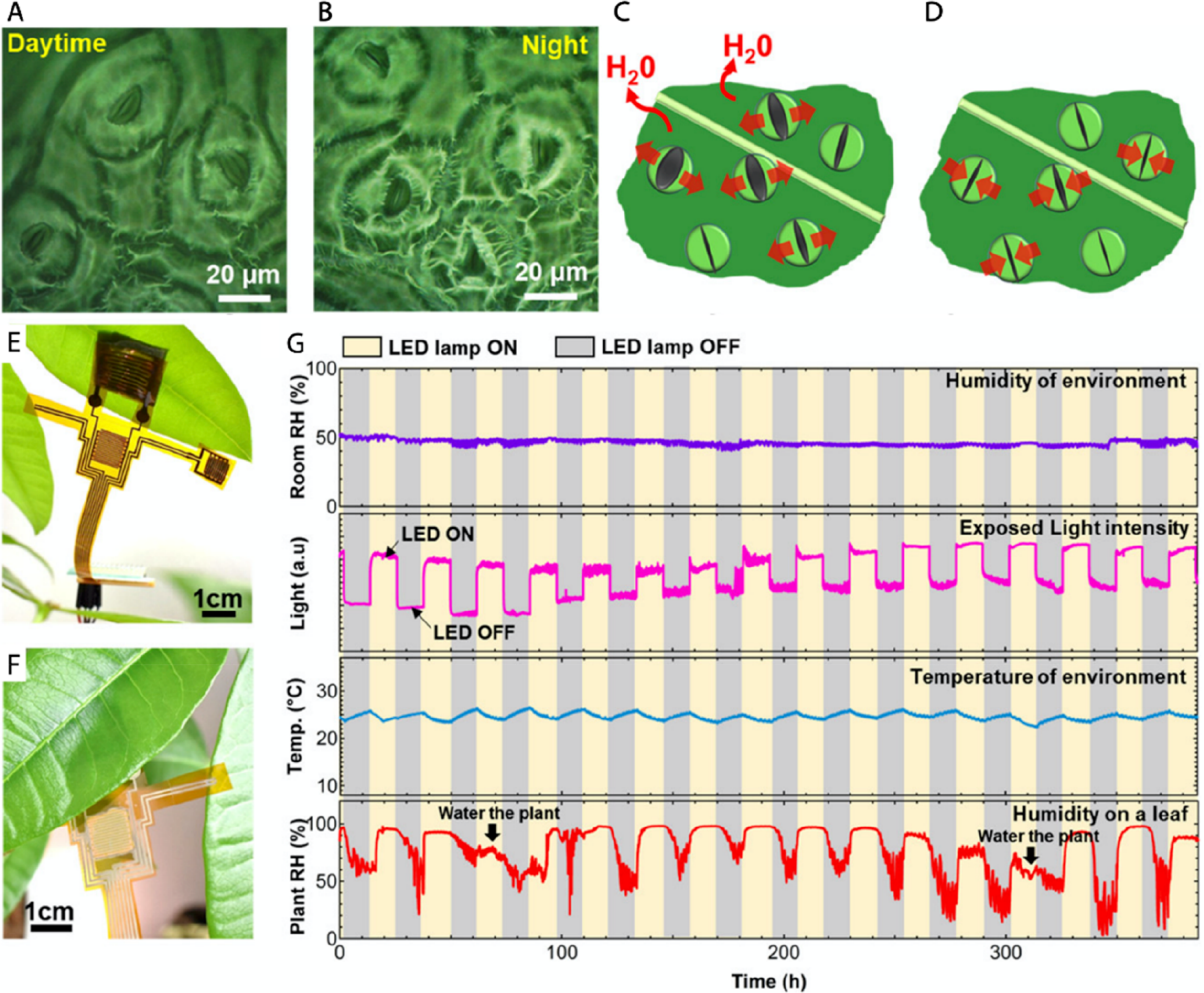
Monitoring of plant transpiration and ambient conditions using a wearable sensing platform. (A) Optical microscopic images of leaf stomata that open in daytime and (B) close in nighttime. (C) Depiction of plant transpiration process where water is released through open stomata, resulting in higher leaf humidity. (D) Close stomata prevent water loss reducing leaf humidity. (E) Back- and (F) front-side photography of the device attached to bottom side of leaf, where module for transpiration monitoring is attached to the leaf, while environmental sensors are suspended in air. (G) Environmental parameters and plant humidity monitored using the developed sensing platform. Reprinted from ref (21). Copyright 2020 American Chemical Society.

Instead of following single stomata dynamics, other electronic methods for determining transpiration rely on monitoring humidity changes on the leaf surface that arise from the stomata activity and not the ambient changes. In the majority of plants, the stomata density is higher on the lower epidermis to reduce plant water loss; thus, all RH sensors described below were attached on the adaxial side of the leaf. A polyimide-based RH sensor was used to monitor plant transpiration, where the polyimide film on sticky polyethylene terephthalate (PET) substrate was attached to tobacco (*Nicotiana tabacum*) leaf with the RH sensing layer facing the air.^37^ The device demonstrated significant increase of the polyimide capacitance after plant watering followed by an abrupt decrease the next day. A processing circuit with a Bluetooth module allowed for monitoring the plant water status using a mobile device. To enable better monitoring of transpiration without ambient humidity interference, the articles described below positioned the sensors with the RH sensing layer facing the leaf. By leaving an air gap between leaf and sensor they also prevented the accumulation of water released by stomata that could affect plant transpiration. Oren et al. engineered a low-cost, scalable, and roll-to-roll method for patterning and transferring graphene-based nanomaterials onto tape to fabricate flexible RH microsensors.^38^ The sensing mechanism relied on a humidity-dependent modulation of the graphene electrical resistance. The device was attached to a maize leaf with a 170 μm thick air gap between leaf and device. By attaching multiple RH sensors on different leaves, the water transport along the stem could be tracked, demonstrating different water transport dynamics between two maize variants. Such tests might help to select species of more efficient water transport in breeding. Lan et al. developed a flexible capacitive-type RH sensor based on graphene oxide layer on top of interdigitated graphene electrodes.^39^ This device shows one of the highest RH sensitivity reported so far (3215 pF per% RH), low hysteresis and a long stability. The system was attached to *Epipremnum aureum* leaf with gap enabling real-time monitoring of gradual reduction of stomata conductance during few days of drought stress. In contrast, the humidity sensor that was attached on the leaf with the active side facing the air showed an abrupt reduction in leaf humidity readout already 12 h after watering^37^ while typically the plant transpiration gradually decreases in drought conditions. The multimodal system for microclimate monitoring developed by Lu et al. (discussed in the section 2) also has a module for real time probing of the plant hydration status (Figure 5).^21^ A ZnIn_2_S_4_ nanosheet-based humidity sensor stuck to a leaf with 2 mm thick air gap allowed to observe stomata opening induced by light illumination and gradual plant dehydration in drought conditions. By attaching several sensors on different leaves along the plant, it was possible to observe that the leaves closer to the root had higher humidity faster than top leaves.

### 3.2 Plant Growth

Plant growth can be monitored electrically via wearable stretchable resistive sensors whose resistivity changes with strain. The sensor should be attached at the growing part of the plant so when the plant tissue elongates, the resistance of the sensor will change. Nassar et al. developed stretchable strain sensors based on the buckling technique where a gold layer was deposited on a thin prestretched PDMS substrate forming a wrinkled gold layer that would sustain strain.^16^ The elongation of a barley leaf was monitored for 2 h, showing a total growth of 285 μm with an exponential growth trend. The sensor also monitored bamboo stem growth for 24 h for two different days, revealing an average elongation of 905 μm day^–1^.

Growth sensors can be used to monitor fruit development as well. Such a stretchable sensor was fabricated by simply depositing carbon nanotubes with a graphite ink on latex glove substrate, resulting in a low-cost device.^40^ The sensor was attached to the fruits surface using double-sided tape and integrated with a readout system to monitor the fruit growth for 9 days. The continuous growth monitoring indicated that eggplant (*Solanum melongena L.*) and pumpkin (*Cucurbita pepo*) fruits grow at an average rate of 3 and 5.9 μm min^–1^, respectively, with a faster growth during nighttime. Furthermore, this low-cost plant wearable sensor revealed that eggplant fruits grow in a stepwise manner: ∼10 s intervals of fast grow are separated by ∼10 s intervals of rest. Such oscillatory growth behavior was previously observed only for micron-size plant tissues such as pollen tubes^41^ and plant root hairs^42^ using optical microscope and time-consuming data analysis.

A smart system for self-powered light stimulation of plants and sensors for monitoring plant growth and environment has been recently developed by Hsu et al.^43^ Most of the active components were based on a poly(acrylic acid) (PAA) electrolyte with reduced graphene oxide nanofiller (RGO) and polyaniline coating (PANI). The unique formulation of the PAA-RGO-PANI hydrogel resulted in a composite system with multifunctionality. The self-powered light stimulator consisted of a triboelectric generator, supercapacitors and LEDs. A freeze-dried PAA-RGO-PANI hydrogel was combined with a polyimide film to form the tribogenerator (for details on working mechanism see section ‘8.2. Triboelectric nanogenerators’) enabling harvesting of acoustic energy, rainfall, and wind energy with a power output of 424 mW m^–2^, outperforming other triboelectric clean energy harvesters reported so far. The generated current was stored in five supercapacitors based on freeze-dried PAA-RGO-PANI for powering 20 LEDs to stimulate plant growth (“light fertilizer”). Stimulating the plants with self-powered LEDs for 3 h per day, 18 days in a row, significantly increased the growth rate of *Aloe vera* leaves and pepper fruits. To monitor the growth rate, the PAA-RGO-PANI hydrogel was molded to form an elastic band for pepper fruits or patterned as interdigitated electrodes on a PDMS substrate to form a thin wearable device for *Aloe vera* leaves. The growth sensor showed a linear resistance increase during the induced strain changes, exhibiting a wide detection range up to 200% strain along with a high sensitivity (gauge factor: 4.5). However, the growth rate was measured only once per day and only as relative change. Furthermore, the PAA-RGO-PANI hydrogel was used as an ammonia sensor. When the hydrogel was exposed in various concentration of ammonia gas, the resistance of the hydrogel increased. It was hypothesized that adsorbed ammonia gas generates protonated ammonium cations on PANI that induce a chemical dedoping and therefore an increase of the resistance.

To the best of our knowledge, the strain sensor based on the nanocomposite with polyaniline is the only example that uses conjugated polymers for monitoring plant growth.^43^ In health monitoring, conjugated polymers-strain sensors receive an increasing attention for monitoring human body movement.^44^ Upon strain application, conjugated polymers show an increase of resistance due to the disruption of the conductive network. Multiple wearable PEDOT-based strain sensors have been developed, using PEDOT:PSS/poly(vinyl alcohol) on PDMS,^45^ PEDOT:sulfonated lignin hydrogel,^46^ PEDOT/poly(vinyl alcohol) hydrogels,^47^ PEDOT:PSS/sodium alginate composite fibers^48^ and PEDOT:PSS/poly(vinyl alcohol) fibers.^49^

### 3.3 Bioimpedance Spectroscopy

**Figure 6:**
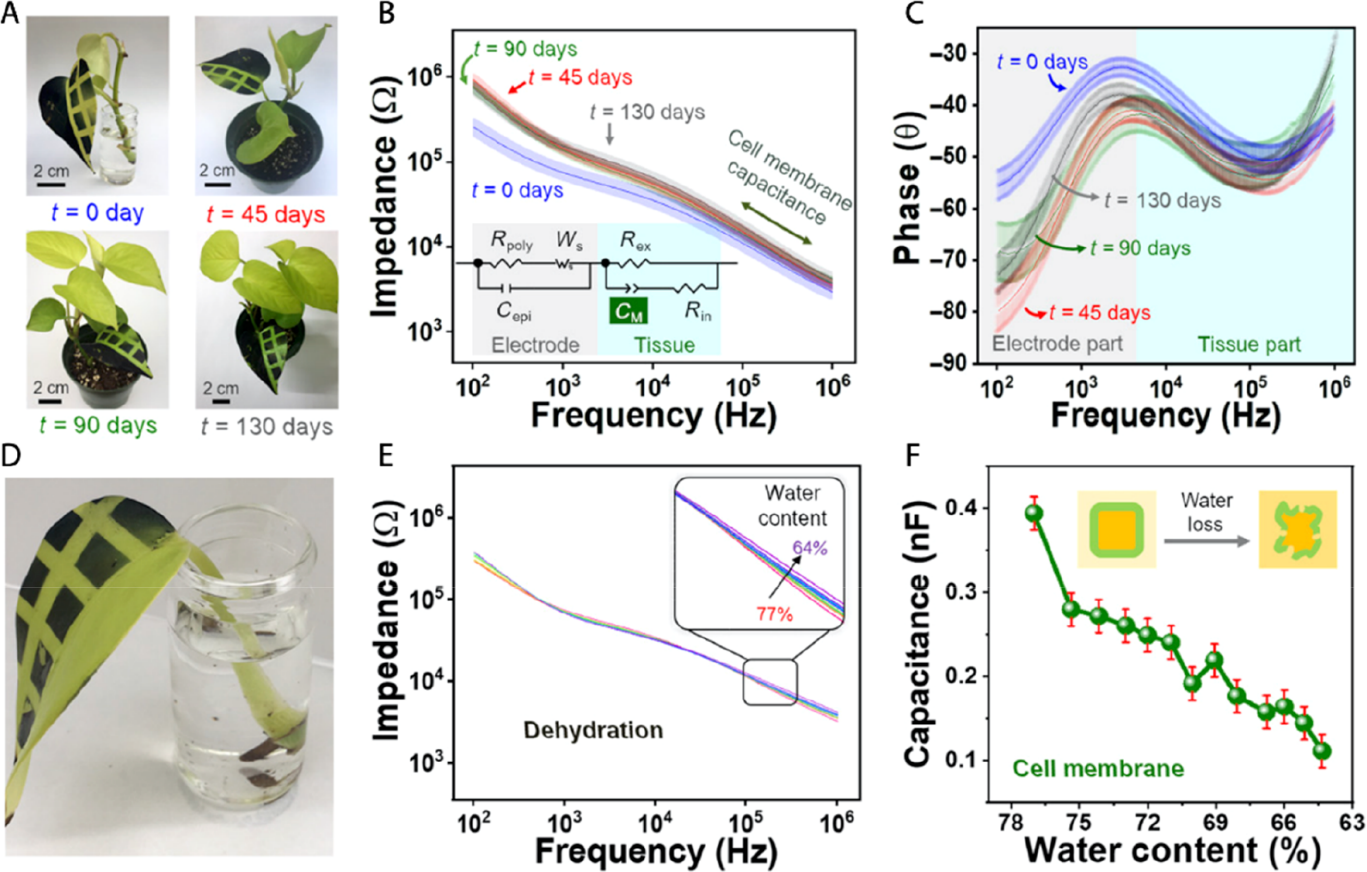
Vapor-printed polymeric tattoos for plant health monitoring based on electrochemical spectroscopy. (A) Photography of PProDOT Cl tattoo deposited on pothos seedling that was transferred to soil for monitoring of the plant response and device stability over 130 days. (B) Impedance and (C) phase response of the tattoo collected within 13 days of the stability test. At the plant diagnostic window above 10^3^ Hz, the sensor response is not affected by its age. (D) Photography of the leaf that was used for drought experiment. (E) Impedance response and (F) calculated cell membrane capacitance of the tattoo during leaf dehydration. Reprinted from ref (54). Copyright The Authors, some rights reserved; exclusive licensee AAAS. Distributed under a CC BY-NC 4.0 license (http://creativecommons.org/licenses/by-nc/4.0/).

Electrochemical impedance spectroscopy (EIS) is widely used in plant research to characterize cellular structure and moisture content giving information on plant health and quality, e.g. for early detection of pathogen infection,^50^ salinity stress,^51^ and fruit ripening.^52,53^ EIS in plants is typically performed using metal needles that pierce into plant tissue, which damage the test site and may induce necrosis. Alternatively, epidermal conducting patches can be applied; however, they may suffer from delamination, inhibiting long-term measurements and increasing the limit of detection.^54^ To address this problem, Kim et al. developed a technique to pattern conductive polymer films directly on plant leaves by vapor-phase polymerization (VPP), forming conducting polymer tattoos (Figure 6).^54^ Small seedlings or detached leaves were placed in a quartz reactor that contained the 3,4-propylenedioxythiophene (ProDOT) monomer and oxidant FeCl_3_. Monomer and oxidant were heated, forming vapors which led to the oxidative polymerization and deposition of a conjugated polymer layer. Polyimide tape was used as a mask to create the electrode pattern on the leaves. VPP requires the plant to be exposed to mild vacuum (1000 mTorr range). However, neither the mild vacuum nor the polymer coating induced any observable effects on the pothos seedlings growth characteristics or chlorophyl content (tested up to 45 days from the coating). The VPP-deposited PProDOTCl electrode also did not block stomata as concluded via microscopy from any tested detached leaf models and showed good surface adhesion and mechanical properties in bending tests. PProDOTCl tattoos were then used for bioimpedance spectroscopy on the pothos leaf. The impedance of the leaves remained unchanged over 130 days in the diagnostically relevant frequency range (frequencies above 10^3^ Hz). In order to extract tissue specific parameters from the impedance spectra, the authors fitted the data into an equivalent circuit model that accounts for the resistive and capacitive elements of both electrode and plant tissue. To demonstrate the possibility of using the tattoos for drought monitoring, the authors studied how the impedance of the leaves changed when the leaves were artificially dried. They found that the 13% decrease in leaf water content results in significant decrease in the cell membrane capacitance and extracellular fluid resistance. This was attributed to the increased ionic concentration within the leaf. In a following work, the conductive tattoos were applied for impedance-based early detection of ozone damage in grape leaves. The authors first compared the performance of organic electrodes PEDOT-Cl deposited by VPP and PEDOT:PSS deposited by drop casting with typical inorganic conductors, such as silver and graphite. The polymer tattoos showed better biocompatibility than the inorganic electrodes without any visible damage in the leaf tissue while graphite and silver showed little or severe damage as defined by visual inspection. VPP-deposited PEDOT-Cl films adhered strongly to the leaf surface without observable cracking or delamination, evaluated for 18 months in growing grape plant. In contrast, the solution-deposited electrodes, i.e., silver, graphite and PEDOT:PSS adhered poorly to the leaf tissue, resulting in cracks during the bending test. They also did not sustain rinsing with tap water. Furthermore, the conductivity of PEDOT-Cl film was not significantly affected by ozone exposure, most probably due to its relatively high crystallinity that reduces ozone penetration. As such, PEDOT-Cl electrodes were chosen for monitoring of tissue damage upon ozone exposure in detached grape and apple leaves. Upon ozone exposure, PEDOT-Cl tattoos had a characteristic dose-dependent increase in impedance and phase signal at 10^5^ Hz with a limit of detection of 10 ppmh, which is below the estimated dose that affects fruit production yield. In a later work, the PEDOT-Cl tattoos were used for single-frequency bioimpedance analysis for UVA damage in detached host leaves. VPP polymer electrodes are a promising solution to monitor various stresses; however, their application in intact plants is still to be demonstrated. Furthermore, the need of a vacuum chamber for the deposition constrains the use of these electrodes to laboratory environment with small samples such as detached plant tissues or small seedlings.

### 3.4 Ionic Content

Many important processes in plant biology are defined by ionic signaling. Therefore, monitoring the changes of ionic concentration within the plant or at the soil interface can give valuable information for plant biology fundamental research, for understanding salt tolerance mechanisms,^55^ and for optimizing soil fertilization^56^ for breeding purposes. Conventionally, ion analysis in plants has been performed using inductively coupled plasma atomic emission spectroscopy, atomic absorption spectroscopy, or mass spectrometry, which require drying and grinding the tissue inhibiting dynamic monitoring in intact plants. Ion-selective electrodes (ISE) translate ion concentrations directly into electrical potential changes,^57^ and they are widely used in biological analysis, offering a simple and reliable low-cost method. The most basic structure of ISE consists of an electrode combined with an ionophore that allows only specific ions to reach the electrode. Traditional ISE are based on liquid ionophores and electrolyte; however, liquid-based ISE are fragile, require maintenance, and impose challenges for miniaturization.^58^ To overcome these issues, ionophore-doped polymeric membranes were developed, offering easier processability and a wide range of ion selectivity, detecting nearly 100 different analytes.^57^ To completely eliminate the use of liquid components and enable solid-state ion-to-electron transduction, various conjugated polymers and several nanomaterials have been successfully applied, resulting in all solid-state ISE of a significantly improved robustness and reliability, and from which the need for maintenance is largely eliminated. Furthermore, solid-state devices can be easily manufactured using standard microfabrication techniques, allowing their miniaturization for better integration in plant systems. An excellent recent review, summarizing the advances in solid-state ISE, can be found in ref (57), while this section will highlight recent ISE application for ion analysis in intact plants, focusing especially on conjugated polymer-based ISE.

**Figure 7:**
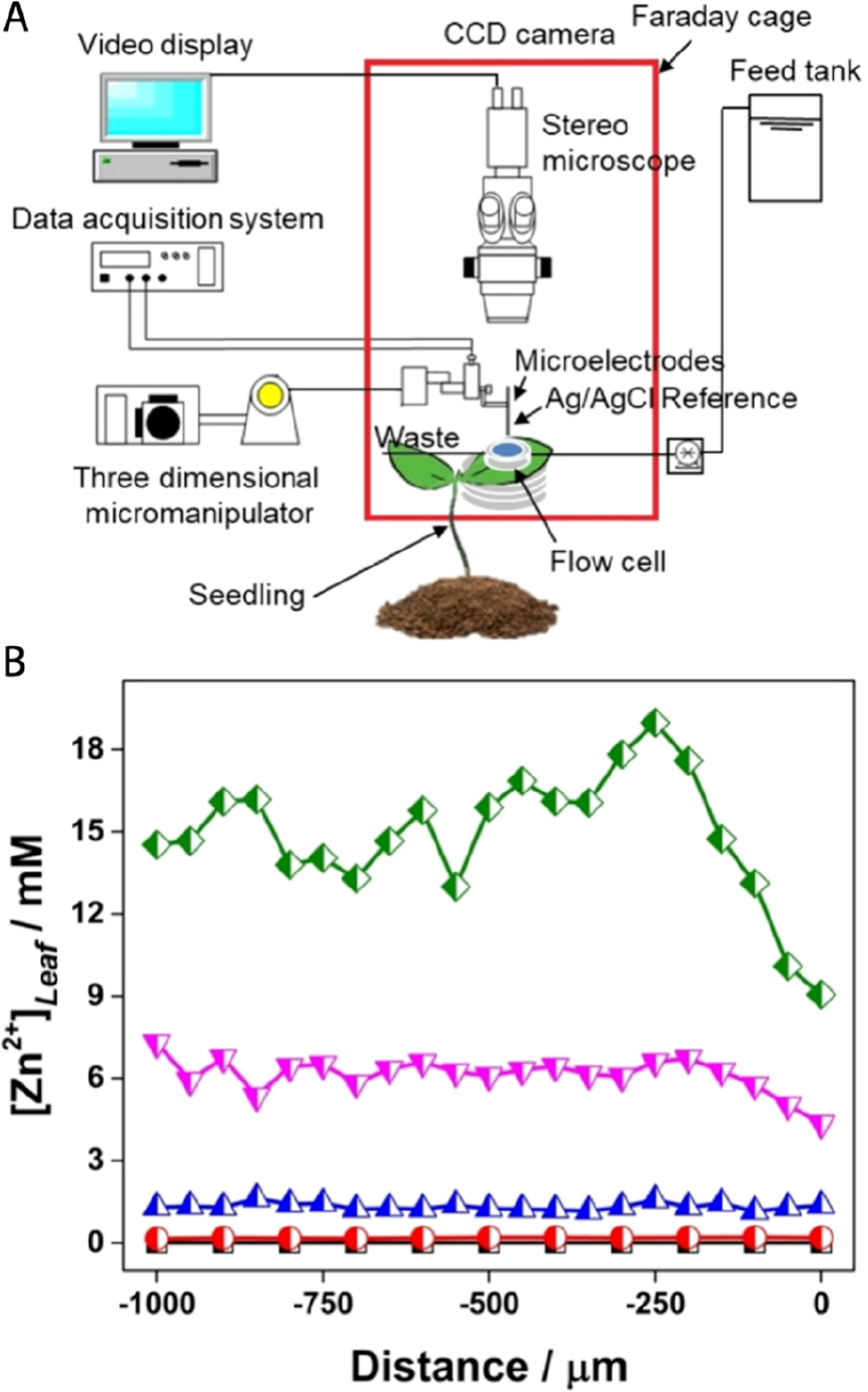
Quantification of Zn^2+^ fluxes in leaves and roots of citrus tree using a miniaturized ion-selective electrode (μ-ISE) based on the ionophore-doped polymeric membrane and conducting polymer POT as solid ion-to-electron transducer. (A) Depiction of experimental setup: the leaf or root was exposed to a Zn^2+^ solution using a flow cell while ion fluxes in tissue were determined using ISE and microelectrode ion flux estimation (MIFE) technique. (B) Zn^2+^ concentration in the flow cell as a function of distance from the orange tree leaf for various bath concentrations (black squares: 0 mM, red circles: 0.20 mM, blue triangles: 1.3 mM, pink triangles: 6.0 mM, green rhombus: 15.2 mM Zn^2+^) determined by developed μ-ISE. The results indicate that Zn^2+^ uptake by the leaf occurs mainly by passive diffusion. Reprinted with permission from ref (59). Copyright 2018 Wiley-VCH Verlag GmbH & Co. KGaA.

Sulaiman et al. used an all solid-state ISE to analyze the stress-induced calcium signaling in *Arabidopsis thaliana*.^58^ The device consists of PEDOT-coated carbon fiber electrode within a glass micropipette filled with a cocktail of solid-state Ca^2+^-selective membrane. With this ISE, changes in calcium concentration at the root proximity were monitored over time. Ca^2+^ release was observed only upon rapid cooling, while a gradual temperature decrease did not elicit any observable changes. A Zn^2+^-selective ISE based on a Zn^2+^ ion selective polymeric membrane and a metal wire coated with the conducting polymer poly(3-octylthiophene-2,5 diyl) (POT) as solid ion-to-electron transducer was developed by Church et al. (Figure 7),^59^ reaching a detection limit of about 4 × 10^–7^ M. The sensor was applied to determine Zn^2+^ transport processes in the foliage and roots of citrus trees. The leaf or root was exposed to a Zn^2+^ solution using a flow cell while ion fluxes in tissue were determined using a microelectrode ion flux estimation (MIFE) technique. The ISE electrode was moved in a direction perpendicular to the leaf and root surface from 1000 to 50 μm above it to prevent electrode damage. The results indicate that the Zn^2+^ uptake in roots is higher than in the leaves due to their intrinsic ability to absorb nutrients, while the Zn^2+^ uptake by both organs was shown to occur mainly by passive diffusion. The developed tool gave promising characteristics for ion uptake studies to evaluate the effect of nutrient therapy on plant disease mitigation. Miah et al. developed a disposable needle-type ISE for analysis of Na^+^ and K^+^.^60^ The device was inserted in different parts of rice plants (roots, stem base, stem, and leaves) and determined the Na^+^ and K^+^ concentrations that were in good agreement with atomic emission spectrometry results.

On the other hand, Coppedé et al. used a yarn-based organic electrochemical transistor (OECT) for monitoring ionic concentration changes in the stem of a tomato plant. Typically, the OECT has a conjugated polymer-based channel whose conductivity is modulated via a gate electrode. The gate and channel are coupled via an electrolyte, making this device particularly attractive for bioapplications.^61^ In this work, the transistor channel consisted of a PEDOT:PSS-coated cotton fiber while a thin silver wire acted as the gate electrode.^62^ PEDOT:PSS is a doped conjugated polymer where the charges on PEDOT backbone are compensated by the sulfonate groups on the PSS. Therefore, in the absence of gate voltage the channel current of the OECT is high. When positive voltage is applied at the gate electrode in respect to the channel, PEDOT:PSS is dedoped as cations from the electrolyte migrate into the channel and compensate the PSS dopant moieties.^63^ The normalized transistor response followed the circadian rhythm of the plant for over 42 days with an increased modulation during nighttime. It was hypothesized that the change in the transistor response was due to a higher electrolyte content in the sap during nighttime and, therefore, larger modulation of the transistor current due to more effecting gating. After 42 days of implantation, the presence of necrotic cell layer around the metallic gate was observed while the overall morphology of the stem was not affected. In following works, the silver-based gate was replaced by PEDOT-functionalized thread to reduce necrosis around the insertion point. The thread-based PEDOT OECTs were used for monitoring vapor pressure deficit^64^ and drought^65^ based on the changes of the sap electrolytic content under these conditions. In both cases, the normalized transistor response was decreasing gradually with the stress duration. This behavior was attributed to the accumulation of ions in the sap due to reduced transpiration rate in stress condition. As such, the method enabled to detect the drought stress in plants within the first 30 h of water deprivation. However, information such as the contribution of each ion to the signal as well as the measured range of ionic strength are not clear. The thread gate and channel were inserted along the whole stem diameter with no encapsulated part therefore it is not clear which plant tissues were contributing to the transistor response.

### 3.5 Metabolite Monitoring

Biochemical sensors have received a lot of attention for Point-of-Care applications, enabling, for example, millions of people to manage diabetes by using an everyday glucometer based on electrochemical glucose sensors. However, biochemical analysis in plant biology is usually performed with mass spectrometry or enzymatic assays on collected tissue samples. Although these methods are reliable and can have a very low detection limit, they cannot be performed in vivo and in real time. Another method commonly used in plant biology is genetically encoded biosensors where the plant is modified to express or quench a fluorescent signal in the presence of the biomolecule of interest. While these methods can have subcellular resolution, the detection relies on the use of a fluorescence or even confocal microscope restricting the application to tissues that can be visualized under a microscope and hindering any application to field conditions.

Our group developed enzymatic sensors based on the organic electrochemical transistor for monitoring sugars in in vitro^66^ and in vivo^67^ plant systems. Sugars are important biomolecules in plants as they are the energy source but also signaling molecules involved in gene expression and stress responses. OECT are attractive devices for biosensing as they can amplify the signal, enabling the miniaturization of devices with a high signal-to-noise ratio.^68^ The OECT can be converted to an enzymatic biosensor through gate functionalization and has been shown to allow an efficient detection in complex biological media. When the analyte is present in the solution, an electrochemical reaction takes place at the gate, which becomes amplified through the modulation of the channel current. The size of the active sensing area can reach single plant cell resolution via microfabrication.

Chloroplasts are the plant organelles responsible for photosynthesis. During the day, sugars are synthesized and their excess is stored at the chloroplasts in the form of starch granules. During the night, the starch granules break down to glucose and maltose that are then exported to the cytosol, converted to sucrose, and transported via the vascular tissue to more distant tissues. We presented an OECT glucose sensor that could detect in real time glucose export from chloroplasts.^66^ A planar OECT was fabricated on a polyethylene naphthalate (PEN) substrate with a PEDOT:PSS channel and a Au/PEDOT:PSS gate that was functionalized with glucose oxidase and Pt nanoparticles to acquire glucose selectivity. More specifically, when glucose is oxidized by glucose oxidase, H_2_O_2_ is generated. H_2_O_2_ is then oxidized at the gate electrode, catalyzed by the Pt nanoparticles. The electron transfer at the gate electrode induces an increase in the effective gate voltage that results in a change in the transistor current. Chloroplasts were isolated from the plant in two distinct metabolic phases, sugar biosynthesis and starch degradation mode. The glucose sensor was simply immersed in the isolated chloroplast solution in a vertical configuration, and it monitored over time the glucose export from the chloroplasts. Glucose was detected only from chloroplasts that were isolated from the plant during nighttime in agreement with the current understanding of starch degradation. The sensors provided quantitative data for glucose export with a time resolution of 1 min, while reports in the literature that rely on enzymatic assays showed a time resolution of 30 min. The direct coupling of the chloroplasts with the biosensor enables monitoring of the initial export kinetics that is not possible with enzymatic assays, due to the lag time required for sample preparation.

**Figure 8:**
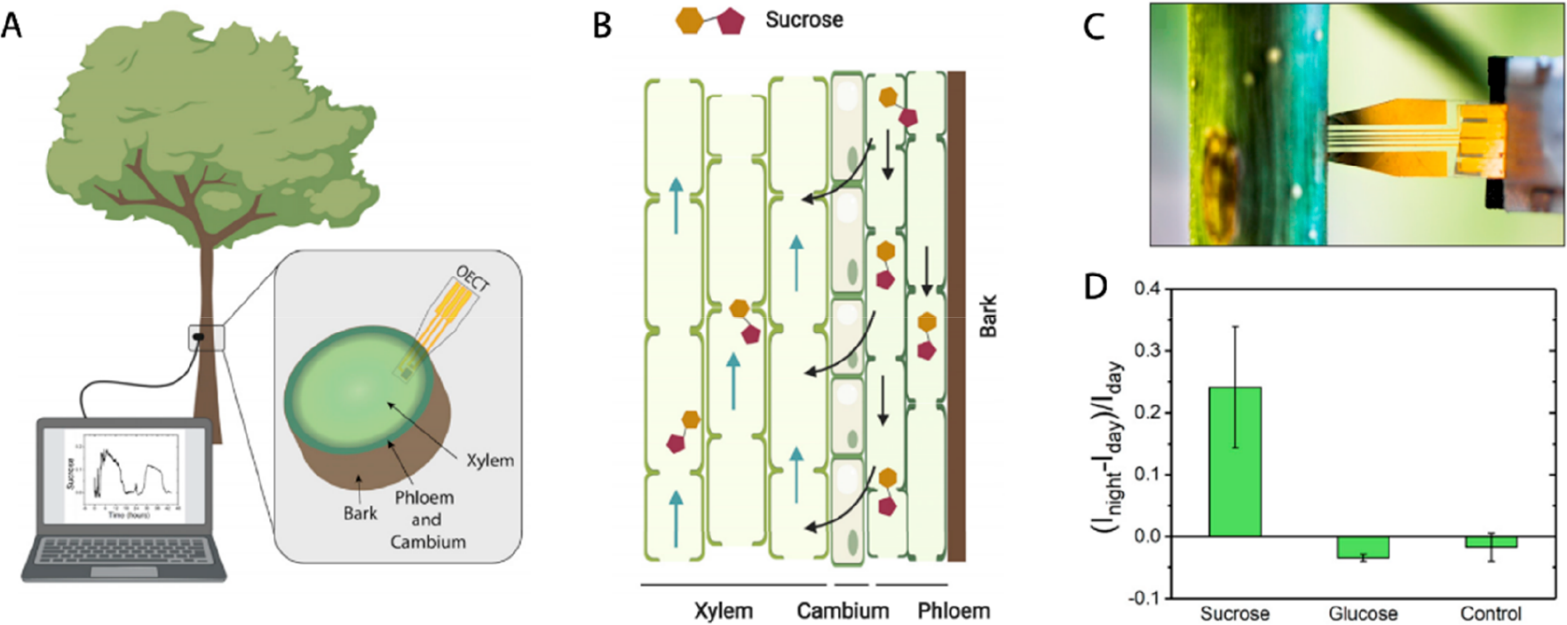
Implantable organic electrochemical transistor (OECT) for in vivo monitoring of sugars fluctuations in the xylem. (A) Schematic representation of biosensor inserted in the xylem tissue of stem of a young poplar tree. (B) Illustration of the sucrose transport in the vascular tissues: sucrose is mainly transported in phloem from where it is unloaded to xylem to be distributed via transpiration stream. (C) Photograph of the device inserted in a poplar stem. (D) OECT-based sensors enabled unprecedented monitoring of day/night fluctuations of sucrose and glucose in the xylem sap over 48 h. Reprinted with permission from ref (67). Copyright 2021 Cell Press.

In a following work, we presented glucose and sucrose OECT sensors for real time monitoring of sugar variations in the vascular tissue of a Hybrid Aspen tree (*Populus tremula x tremuloides*) (Figure 8).^67^ Sucrose does not have a corresponding enzyme to oxidize it. Therefore, to detect sucrose electrochemically, we functionalized the gate electrode with three enzymes: invertase, which hydrolyses sucrose to glucose and fructose, mutarotase, which converts α-d to β-d glucose, and glucose oxidase, which oxidizes glucose. The OECT sensors were inserted in the stem with their active site localized in the mature xylem tissue and recorded continuously over 48 h with the use of a portable low-cost Arduino device. Microscopy analysis revealed that, after 2 days of implantation, no significant wound response from the plant was evoked while 5 days of implantation initiated the formation of cork tissue that could eventually isolate the device from the tissue of interest. Although the sensor footprint had dimensions of 125 μm × 1 mm × 3 mm, the insertion into the hard xylem tissue required an initial incision with a scalpel; therefore, further engineering on the insertion method could minimize the wound response. The sensors revealed diurnal variations of sucrose in xylem tissue with sucrose concentration increasing during nighttime and decreasing during daytime. On the other hand, glucose concentration remained constant, while control devices showed no variation in their response. For comparison, we performed an ex vivo xylem sap analysis with enzymatic assays. The ex vivo analysis showed that sucrose, glucose, and fructose concentration increased during nighttime. We believe that this difference arises from the highly invasive collection of the sap for the ex vivo analysis where the plant is decapitated, the phloem is removed, and then the sap is extruded via the root pressure over 1 h. Likely, during this process, the cells are injured and therefore contributed to the collected fluid. Furthermore, wounding activates cell wall invertases that breakdown sucrose to fructose and glucose. Our study revealed a diurnal dependence of the sucrose concentration in the mature xylem that was not observed before and is likely to be linked with metabolic and/or physiological processes such as growth rate and starch degradation. One limitation of this technology is that it can be used only for qualitative observations as quantification is hindered by the unknown initial concentration of the analyte in the in vivo environment. Further engineering on the sensors design is required to enable quantitative monitoring, for example, integrating an internal reference.

## 4 Devices for Modulating Plant Physiology

Plant hormones (phytohormones) are signaling molecules that are present in trace quantities (ng to pg g^–1^ fresh weight) and act as major regulators of plant growth and development.^69,70^ For example, auxin is a key regulator of numerous physiological processes in plants, such as cell elongation, differentiation phototropism, gravitropism, apical dominance, fruit development, and abscission,^71^ while jasmonates activate plant defense responses to elicitors. Abscisic acid, on the other hand, regulates plant drought responses. In agriculture, for many years, external plant hormones have been widely applied to increase the cultivation yield, to improve the quality of the harvests, and to increase plants’ resistance to disease or stress. However, further improvement of food production yield, most needed in a projected warmer and drier climate, depends on a better understanding of hormone biosynthesis and signaling as many crucial questions remain unanswered.

Exogenous hormone application is an important experimental technique for understanding how hormones orchestrate plant growth and development.^72,73^ Furthermore, many plant physiological processes and stress tolerance can be significantly modulated using exogenous hormone delivery.^74^ In research settings, hormones are applied by foliar spray, coating, or root soaking. Plant barrier tissues (such as cuticle and epidermis), however, significantly affect the hormone penetration, so at the end an unknown hormone concentration reaches the plant internal tissue hindering quantitative studies. In many cases, detached plant tissues are incubated in hormone solutions, as with epidermal strips, while petiole feeding enables transport through the vascular tissue. In these cases, though, the complex signaling network may be disturbed as the tissue is detached from the rest of the plant. An elegant noninvasive approach for hormone delivery in vivo, directly into the leaf apoplast, is nanoinfusion, where the solution is applied within the stomatal pore using a submicrometer-scale pipet.^75^ Although the application is noninvasive, localized, and quantitative, it is labor intensive and affects the optical properties of the leaf surface,^76^ which prevents its application in quantitative fluorescence microscopy. All these challenges impose the need to develop phytohormone delivery methods that enable a precise dose control, high efficiency, and low invasiveness to move further toward the understanding of plant biology and assist plant engineering.

### 4.1 Fluidic Devices

A microneedle-like “phytoinjector” has been shown to precisely deliver wide range of substances into vascular tissue of tomato, tobacco plant, and citrus tree, demonstrating injections of fluorescent dyes rhodamine 6G and 5(6)-carboxyfluorescein diacetate; luciferin/luciferase bioluminescence reagents and live microorganism *Agrobacterium tumefaciens* loaded with gene vector for plants’ genetic transformation.^77^ Owing to the systematic optimization of the silk-based material properties, the implant showed an appropriate mechanical robustness for injection into various plant tissues and a controlled degradability for preloaded biomolecules release. To target specifically the xylem and phloem, the size and the shape of the phytoinjector was designed to match the location of the targeted tissue, enabling a precise and reproducible payload delivery. However, this targeting method is suitable only for plants with a specific anatomy of vascular tissue. The silk based “phytoinjectors” were demonstrated to deliver tens of nanograms of cargo molecules per injector, which is a suitable range for the delivery of phytohormones, micronutrients, small interfering RNA, and self-replicating microorganisms.

Microfluidic devices have become a powerful tool for studying biological processes within a well-controlled environment at high throughput. Because of the device’s small dimensions, the plant local stress responses to numerous stimuli such as nutrients, chemicals, and environmental changes can be studied at low cost, in a multiplexed and automated manner.^78,79^ Owing to the laminar flow regime in microchannels, multiple hormones can be delivered to selected regions simultaneously. Two recent reviews discuss various microfluidic devices for plant biology applications,^79,80^ while here we will highlight microfluidics for local hormone/chemical delivery.

The first demonstration of *Arabidopsis* roots’ survival in a microfluidics device was shown by Meier et al., where auxin was applied locally to induce a local enhancement of the epidermal hair growth.^81^ The “RootChip” has integrated PDMS valves to route the stimuli and inputs for eight *Arabidopsis* roots simultaneously. By delivering squared pulses of glucose, changes in intracellular sugar levels were observed.^82^ In another work, a microfluidic platform for asymmetric treatments (stimulation with different chemicals at either side of root) has demonstrated that exposure to biotic (flagellin) and abiotic stress (high NaCl concentration) triggers calcium signals of different direction and velocity indicating different communication mechanisms.^83^ A vertical microfluidic chip with a hormone concentration gradient generator was able to supply the microfluidic chamber with eight different hormone concentrations using multiple splittings of hormones and diluents.^84^ So far, the applications of microfluidics in intact plants are limited to young development stage of *Arabidopsis* plants, where the whole plant can be introduced into microchannels. The vertical microfluidic chip described above could, for example, sustain *Arabidopsis* growth for 4 weeks.^78^

### 4.2 Electrophoretic Devices

All of the delivery methods described above are based on fluid flow, where the substance of interest is delivered together with the solvent. However, when excessive liquid is delivered into biological tissue it may result in shear stress, local pressure increase, and significant perturbation of native ionic concentrations. On the other hand, in electrophoretic delivery methods, ions are delivered from a source electrolyte to a target (e.g., cells or tissue) via a membrane or capillary channel under the application of an electric field. The ions move via electromigration and electroosmosis, enabling the delivery of ions of interest without significant fluid flow overcoming convective disturbances in the targeted tissue.^85^ Furthermore, the electronic addressing offers advantages of better control of amplitude and frequency of the ionic delivery. Iontophoresis has received considerable attention for noninvasive delivery of numerous therapeutics in dentistry, ophthalmology, otorhinolaryngology, and dermatology, usually with the tissue acting as the membrane.^85−87^ To the best of our knowledge, there are only two examples that demonstrate electrophoretic delivery of ions in plants. Voss et al. delivered several fluorescent reporter dyes into single intact guard cells of tobacco leaf via current injection through a microcapillary.^88^ The same group used the current-injection technique to stimulate single guard cells with abscisic acid (ABA) in intact *Arabidopsis* leaves.^76^ ABA solution was loaded in a microcapillary that was brought in contact with the guard cell wall. By applying a current of −0.8 nA for 20–30 s stomata closure was observed after 1.44 min, while controlled delivery of benzoic acid did not influence the stomata aperture. Interestingly, very local application of ABA triggered a dose-dependent reduction of the turgor pressure only in the guard cell that was in contact with the delivery tip, while the other guard cell from the same stoma remained unaffected. The concentration of delivered ABA ions and their distribution in the tissue was estimated based on the fluorescence imaging of current injection of the fluorescent dye Lucifer yellow.

**Figure 9:**
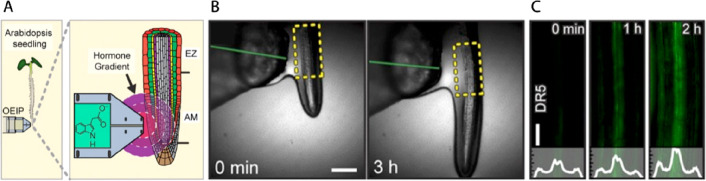
Organic electronic ion pump delivering auxin phytohormone in the proximity of the *Arabidopsis* root. (A) Illustration of the experimental setup highlighting the hormone gradient that was established at the delivery tip of the device. (B) Optical micrographs of the root and OEIP tip indicating that delivered auxin significantly reduced root growth, demonstrating first bioelectronic control of plant physiology. (C) Fluorescence microscope images of the *DR5rev::GFP* reporter which increases its marker fluorescence in the presence of auxin. OEIP-mediated auxin delivery induced increase of the root fluorescence while the effect was higher at the root side facing the device. Reprinted with permission from ref (91). Copyright 2017 National Academy of Sciences.

In conventional electrophoretic delivery devices, the backflow of ions from target to the source complicates quantitative studies and disturbs native ionic concentration gradients in the tissue. To overcome this limitation, Berggren and co-workers have developed the organic electronic ion pump (OEIP), an electrophoretic delivery device where the delivery channel consists of a polyelectrolyte membrane with a high fixed charge.^89^ The OEIP delivery channel connects the source and the target, while the fixed charge of the polyelectrolyte defines the sign of the mobile ions. For example, if the polyelectrolyte has negative fixed charge, then only positively charged ions can be transported from source to target, while the backflow of anions from the target to the source will be hindered due to Coulombic repulsion. The low backflow of ions greatly facilitates the estimation of delivered dose and prevents perturbation of the targets ionic concentration.^90^ However, the presence of the polyelectrolyte membrane limits the size of the ions that can be delivered through its pores.^91^ Traditionally, the OEIP have been used in neuroscience studies both in in vitro and in vivo settings for controlling neural firing,^92^ epileptic seizures,^93^ and therapy,^94^ while the potential to elucidate fundamental questions in plant biology has recently started to be explored. In a first plant application, a planar OEIP was used to deliver auxin in proximity (distance of 100–200 μm) to the root apical meristem of *Arabidopsis* (Figure 9).^91^ The planar OEIP was fabricated on a PET substrate using standard microfabrication techniques such as spin coating, bar coating, and photolithography, enabling the patterning of a 25 μm wide delivery channel that was encapsulated to ensure local ion delivery. To enable delivery of auxin ions, a hyperbranched polyelectrolyte with larger pores size was developed. Operating devices with 1 μA of constant current, the auxin delivery rate was 0.45 ± 0.16 pmol min^–1^. In the roots, high auxin concentration is known to inhibit root elongation and promote lateral roots formation.^71,95^ After 15 min of OEIP-mediated auxin delivery in the *Arabidopsis* root proximity, the inhibition of root elongation was observed, demonstrating the first electronic regulation of the root growth rate. Furthermore, delivering auxin to a mutant reporter plant *DR5rev::GFP* resulted in a significant quenching of the fluorescence indicating uptake and active transport of auxin. Its effect was shown to be stronger on the root side that was closer to the delivery tip, consistently with the estimated auxin gradients. The OEIP-mediated delivery of benzoic acid, a molecule used as a control, had no visible effect on the root growth and the reporter plant fluorescence, verifying that the visible effects were due to auxin solely.

**Figure 10:**
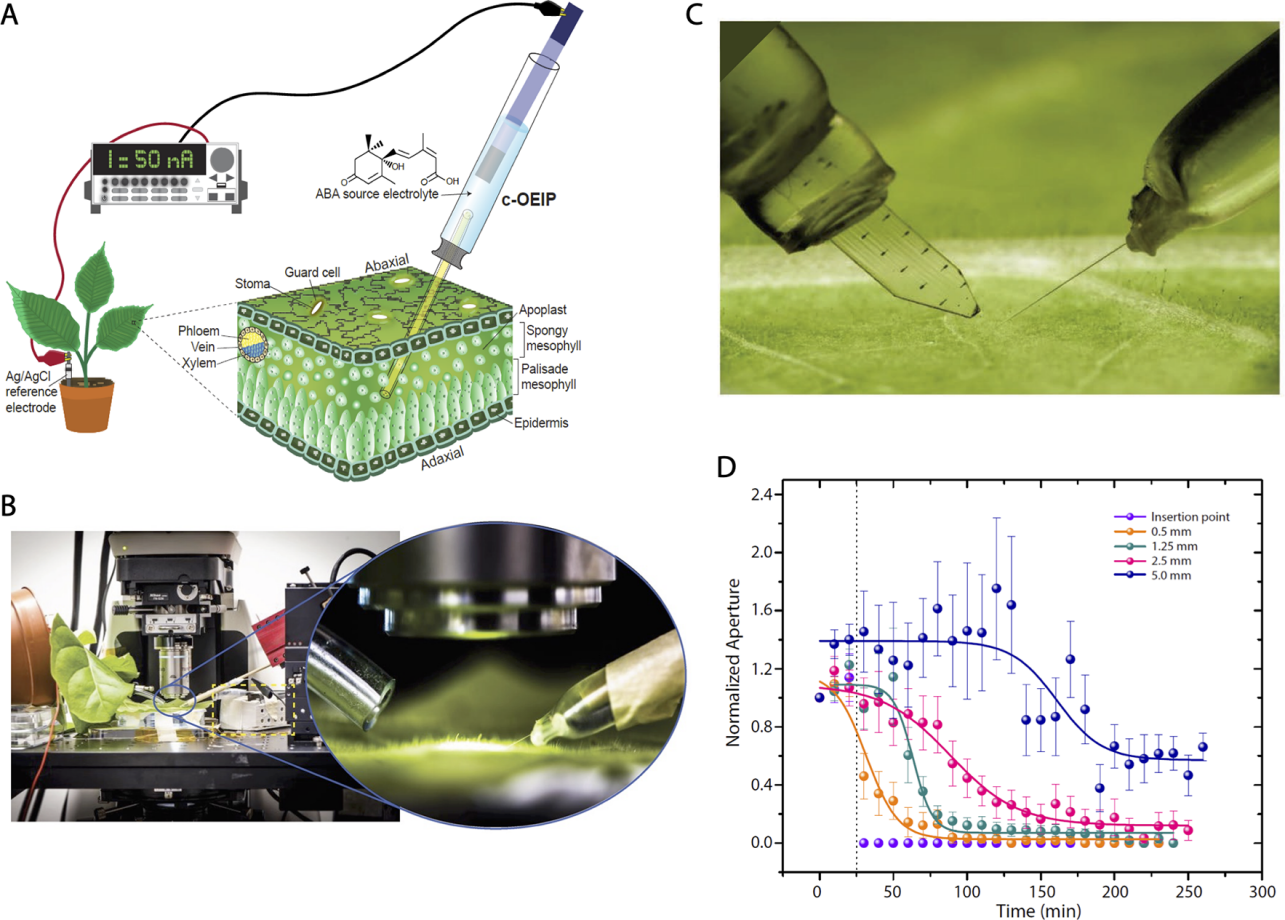
Miniaturized OEIP delivering abscisic acid (ABA) phytohormone into mesophyll of intact tobacco leaf for bioelectronic control of plant transpiration. (A) Schematics and (B) photograph of the experimental setup. (C) Photograph of planar (left) and miniaturized, capillary-based (right) OEIP. (D) Dynamics of the stomata modulation upon OEIP-mediated ABA delivery at different distance from the OEIP insertion point, showing that the delivery triggered sigmoidal, dose-dependent stomata closure with previously unreported signal propagation kinetics. Reprinted with permission from ref,^96^ copyright 2019 WILEY-VCH Verlag.

Taking the work a step further, we recently applied a miniaturized capillary-based OEIP for in vivo delivery of ABA in the apoplast of an intact tobacco plant (Figure 10).^96^ ABA is one of the main hormones involved in plant stress responses and more particularly in the drought response. When plants are under water-deficient conditions, the endogenous ABA concentration significantly increases, inducing stomata closure to prevent further water loss. Although the role of ABA on controlling stomatal closure is evident, still many questions remain unanswered regarding the ABA biosynthesis sources, dose–response, and signal propagation. Conventional OEIP devices have planar geometries that hinder implantation into soft tissue such as leaf. To allow minimal invasiveness during the insertion, capillaries and fibers are often preferred in numerous biomedical applications. As such, we have fabricated an OEIP with a capillary form factor (c-OEIP) by filling with the polyelectrolyte the 20 μm diameter hollow core of the glass fibers (60 μm outer diameter).^97^ The capillary form factor of the c-OEIP enabled the device insertion into the leaf without inducing any significant wound. The leaf tissue conformed smoothly around the OEIP, and even after 24 h of insertion the wound was only equal to the size of the OEIP. The effect of the mechanical insertion of a dry c-OEIP was evaluated based on the stomata response. As stomata are sensitive to environmental changes and mechanical stress, stomata in close proximity to the insertion point (below 0.5 mm) closed by 60–80% when the OEIP was inserted to the tissue but then opened again. The ability of the c-OEIP to deliver ABA was determined using mass spectrometry. The analysis indicated that the use of a hyperbranched polyelectrolyte channel with an optimized addressing protocol resulted in efficient delivery of large cyclic molecules with a rate of 74 ± 19 pmol min^–1^ for an applied current of 50 nA. The OEIP-mediated ABA delivery to intact tobacco leaf triggered the stomatal closure with a spatiotemporal dependence from the delivery tip. After 75 min of delivery, stomata located up to 1.25 mm from the insertion point reduced their aperture up to 100% in a sigmoidal manner, while stomata further away closed only partially, indicating that there is a dose response for ABA. By analyzing the time response of the stomata at various locations from the delivery point, we revealed that the ABA or ABA-induced signal is propagating with constant speed, something that was not observed before. OEIP-mediated modulation of the amplitude of ion delivery and pulsing have also been demonstrated using metal ions, while such modulations using less mobile molecules are yet to be demonstrated.

## 5 Plants as a Chemical Bioreactor

Over millions of years of evolution, plants have developed characteristics that enabled them to survive and thrive in different soils and climates. For example, hyperaccumulators are plants that have adapted to survive in soils with abnormal concentration of compounds that are phytotoxic for the majority of plants.^98^ Hyperaccumulator plants’ ability to uptake and fix toxic compounds in their shoot are used in phytoremediation and phytomining where plants are used to restore polluted lands or mine valuable metals, such as nickel, in a perennial way.^99,100^ Plants additionally developed complex chemical pathways in mild environments to increase their defense mechanism against pathogens, environmental stress, or physical wounding, with the formation of compounds called secondary metabolites.^15^ Key components of their synthesis are enzymes that are nowadays widely used for developing greener synthetic routes of high value chemicals. This section will discuss how living plants’ chemical environment can be leveraged for synthesis of technological materials.

### 5.1 Plant-Mediated Nanostructure Synthesis

**Figure 11:**
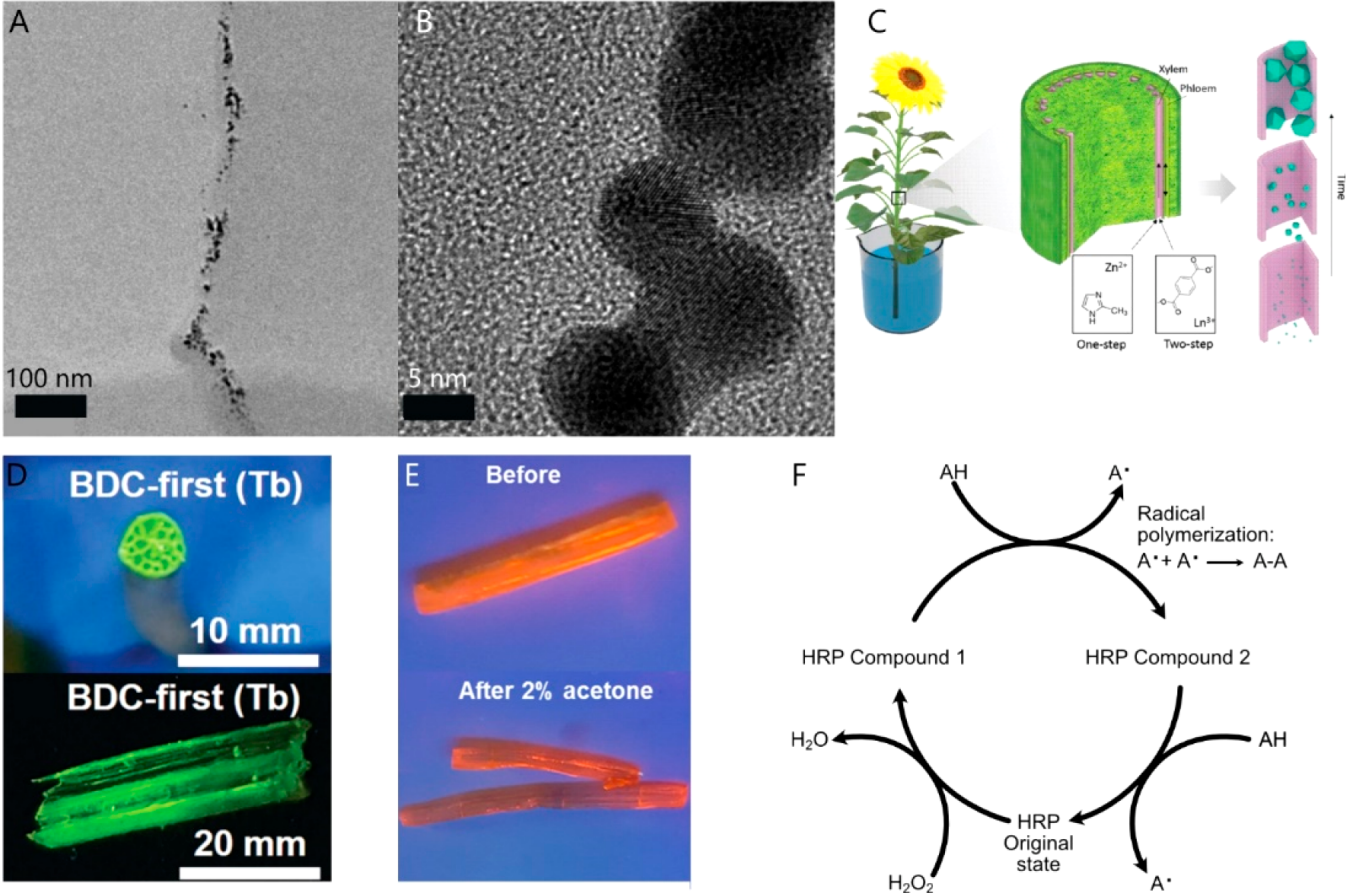
Plant-mediated nanostructure synthesis. (A) TEM image of an array of silver nanoparticles in alfalfa shoot. Scale for 100 nm. Adapted from ref (102). Copyright 2003 American Chemical Society. (B) HRTEM image showing the coalescence of gold nanoparticles inside the plant. Scale for 5 nm. Adapted from ref (101). Copyright 2002 American Chemical Society. (C) Illustration representing the formation of MOFs in plants. (D) Photographs of water lily cross sections under UV light after formation of Tb_2_(BDC)_3_ where the BDC organic ligand was incubated first. (E) Photographs of a water lily augmented with Eu_2_(BDC)_3_ under UV light before and after incubation in 2% acetone. Reprinted with permission from ref (104). Copyright 2017 John Wiley and Sons. (F) Schematic of the peroxidase radical polymerization.

Plants’ uptake and fixation of metals from soil has brought interest to the material science community. By characterizing the structures of metal clusters obtained during the accumulation process, it was found that the plant could be used as a bioreactor for metal nanoparticle synthesis. The first observation was made in alfalfa plants where gold nanoparticles formed in its shoot when the plants grow in an environment rich in Au(III) salts.^101^ Transmission electron microscopy detected Au nanoparticles with sizes ranging from 2 to 20 nm, suggesting a time-dependent particle formation, while nanoparticle aggregates were observed with a size up to 40 nm (Figure 11A). The smallest particles, with an average size of 4 nm, had an icosahedron structure, indicating that the gold atoms were rearranged in their lowest energy configuration structure, even within the plant. Similarly, the formation of silver nanoparticles in alfalfa plants was reported when the plant was grown in an environment rich in AgNO_3_.^102^ The plant reduced the Ag(I) ions into Ag(0) before root uptake and transport to the plant shoot. These particles had a similar size range between 2 to 20 nm, indicating that the plant might limit the particle size (Figure 11B). Additionally, the nanoparticles were found mostly within vascular channels in the plant roots and stem. Only few studies demonstrate the in vivo synthesis of nanoparticles, while most of the studies utilized plant extracts in order to have better control of the reaction conditions and facilitate the particle isolation.^103^ Although these works do not target any specific application of metals nanoparticles synthesized within plants, the emergent field of plant biohybrid systems might incorporate this ability of the plant into device functionality.

Plants also offer a proper environment for self-assembly of metal oxide frameworks (MOFs) through the accumulation of metal ions and organic salts into their vascular tissues.^104^ MOFs structures offer an additional handle for plant tissue modification, opening the possibility for an array of applications, including optical sensing, catalysis, and selective adsorption matrix for gases such as CO_2_.^105^ Richardson et al. demonstrated the assembly of two different MOFs structures within the plant. MOFs such as Zn(MeIm)_2_ have slow formation kinetics that allows the metal ion (Zn^2+^) and the organic salts (MeIm, 2-methylimidazole) to be infused in the plant cuttings simultaneously and then self-assemble into a MOF (Figure 11C). On the other hand, complexes with fast formation kinetics, such as Eu_2_(BDC)_3_ (BDC, terephthalate), and Tb_2_(BDC)_3_, required a two-step process where the plant is first immersed in the organic salt solution that diffuses slowly and then immersed in the lanthanide ionic solution that diffuses faster (Figure 11D). In this way, the self-assembled structure is formed within the plant. Two-step methods were also successfully performed in intact plants by exposing the roots first to the BDC precursor prior to the successive washing and immersion in a Tb^3+^ solution. This enabled the formation of crystalline complexes in the xylem channels and the leaves. In this example, Eu_2_(BDC)_3_ was applied as a chemical sensor as its fluorescence was modulated in the presence of acetone (Figure 11E). The Tb_2_(BDC)_3_(H_2_O)_4_ MOF structure was also assembled in intact plants to induce a visible readout when pollutants are present in the soil where the plant grew. The precursors, sequentially introduced via the roots, were self-assembled into a MOF structure detectable mostly in the plant stem. Their stability was shown for 2 weeks without any effect on the plant. However, the modulation of the MOF fluorescence, either quenched or enhanced, was not specific to one pollutant. This shows that more work needs to be done to increase the specificity of this sensing approach. A mobile application was developed in parallel to facilitate the readout of this plant biohybrid sensor.

### 5.2 Plant Enzymes as Catalysts in the Synthesis of Organic Electronic Materials

Plants’ biochemistry has been also investigated in the field of organic synthesis because of their ability to synthesize active compounds used as drugs and structural materials.^106,107^ Furthermore, there is a great potential for utilizing extracted enzymes as green catalysts in organic synthesis on a small or large scale.^108^ Enzymes are protein complexes that perform highly selective reactions in different pH conditions and temperatures. The selectivity is a prime resource for organic chemists to perform challenging reactions such as asymmetric synthesis or reactions that transfer the chirality from reagents to products.^109^ However, even though enzymes can catalyze a wide range of reactions in vivo, bulk conditions such as temperature, pH or the nature of the solvent can greatly reduce or even inhibit their activity in in vitro conditions.

One of the relevant enzymatic reactions for bioelectronics is the radical-mediated enzymatic polymerization of conducting polymers using oxidoreductase enzymes. These enzymes use either oxygen or hydrogen peroxide as a substrate, and thus, they are called respectively oxidases or peroxidases. In both cases, these enzymes use an electrochemical antenna, also called cofactor, to oxidize the monomeric unit while at the same time they reduce their respective substrate (Figure 11F). Polyaniline, for example, polymerized in mild conditions (pH 3–4) via the horseradish peroxidase (HRP) catalysis of H_2_O_2_. The regeneration of the peroxidase heme cofactor occurs through the oxidation of two aniline units, forming radicals that initiate the polymerization.^110^ The addition of sulfonated polystyrene (SPS or PSS) acted as a template and a dopant leading to the formation of a water-soluble doped polymer. The polymerization of polypyrrole was also demonstrated with HRP in the presence of H_2_O_2_ and a template.^111^ The optimal reaction was obtained in an acidic medium (pH 2), which is unexpected considering that the activity of HRP decreases in low pH.^112^ Polymerization in acidic medium at 4 °C was found to be optimal for EDOT enzymatic polymerization as well templated with polystyrenesulfonate.^113^ The reaction lasted for 16 h indicating that the activity of the enzyme was preserved along this time. The polymerization was successful at RT as well, but the obtained PEDOT:PSS had a 2 orders of magnitude lower conductivity. One explanation for the enzyme stability was that the EDOT monomers with their low water solubility created a biphasic environment where the enzymes were trapped and protected from the acidic water medium. An alternative for performing the synthesis in milder reaction conditions at pH 4 was to add a terthiophene initiator in the bulk. The terthiophene has lower oxidation potential than EDOT monomer and therefore acted as an electron-transfer mediator during the formation of the initial radicals, facilitating the polymer synthesis.^114^

These examples focused on using enzymatic functionality and selectivity for the synthesis of conjugated polymers in vitro. Enzymes, though, can also be utilized in their in vivo environment for the development of biohybrid interfaces by in situ polymerization. In the next section, we will present how to utilize enzymes in their native environment, in the plant, to form biohybrid interfaces.

## 6 Plant-Based Biohybrid Systems

Going beyond materials synthesis, smart materials integration into plants can augment non-native functionality and convert plants into biohybrid devices. Leveraging plant’s natural processes for device functionality opens the pathway for advanced technological concepts using platforms sensibly integrated into their environment.

### 6.1 Self-Organized Electronics

**Figure 12:**
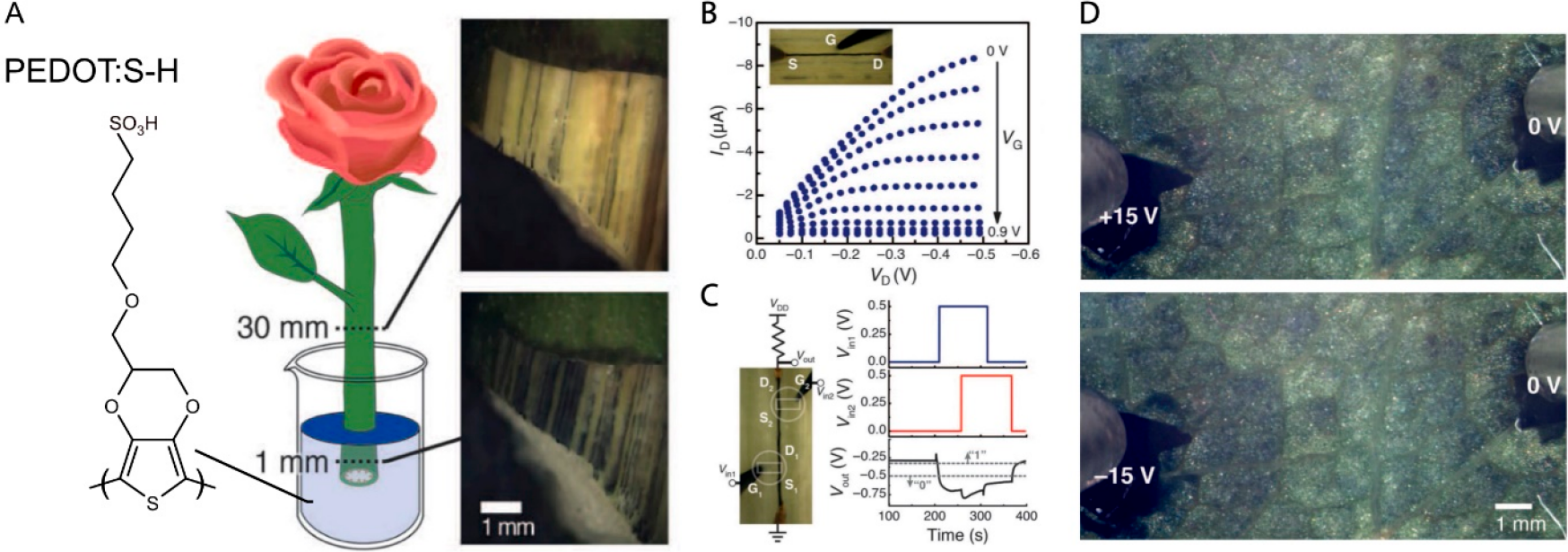
Electronic plants. (A) Schematic of a rose cutting immersed in a PEDOT-S:H aqueous solution with the respective optical micrographs of the conjugated polymer wires at a 30 and 1 mm distance from the immersion point. (B) Xylem-based transistor characterization. Effect of an external gate on the measured current across the source/drain channel. (C) Logical NOR gate manufactured using the PEDOT-S:H xylem and two external electrodes as the two transistors gates. Dashed lines represent the threshold for the logical attribution of 1 and 0. (D) Optical micrographs of vacuum infiltrated leaves with a PEDOT:NFC solution upon the application of +15 V and −15 V. Scale bar for 1 mm. Reprinted with permission from ref (115).Copyright 2015 American Association for the Advancement of Science.

In 2015, we introduced the concept of Electronic Plants where plants are functionalized directly with electronic materials in order to form electrochemical devices and circuits within the plant structure. Organic electronic materials not only are attractive for in vivo functionalization because of biocompatibility and electronic and ionic conductivity but also because they can be processed from aqueous solutions. PEDOT-S:H, a water-soluble and self-doped derivative of PEDOT with a sulfonate group on the monomer, was introduced into the plant tissue.^115^ A rose cutting was immersed for 24 h in the polymer solution; the polymer was uptaken into the plant and self-organized, forming tubular hydrogel wires in the xylem vessels with a conductivity of 0.13 S cm^–1^ (Figure 12A). The conducting wires were then used to fabricate organic electrochemical transistors and a simple digital circuit by having PEDOT-xylem wires acting as transistor channels and gating through the electrolytic medium of the plant (Figure 12B,C). Furthermore, we functionalized the leaf apoplast of the rose with PEDOT:PSS-nanofabricated cellulose (NFC) via vacuum infiltration, a commonly used method in plant biology, and demonstrated electrochromic pixels. As shown previously, PEDOT:PSS organizes along the nanocellulose fibers and therefore can form free-standing electrodes.^116^ By externally applying voltage on the leaf, we could then observe field-induced electrochromism (Figure 12D). The areal electrodes formed within the leaf apoplast were compartmentalized by the veins and formed electrochromic pixels with colors ranging from dark blue for reduced PEDOT and light blue for oxidized. Our work merged, for the first time, organic electronic materials with plant structure and physiology opening the pathway for integrating electronics in living plants.

**Figure 13:**
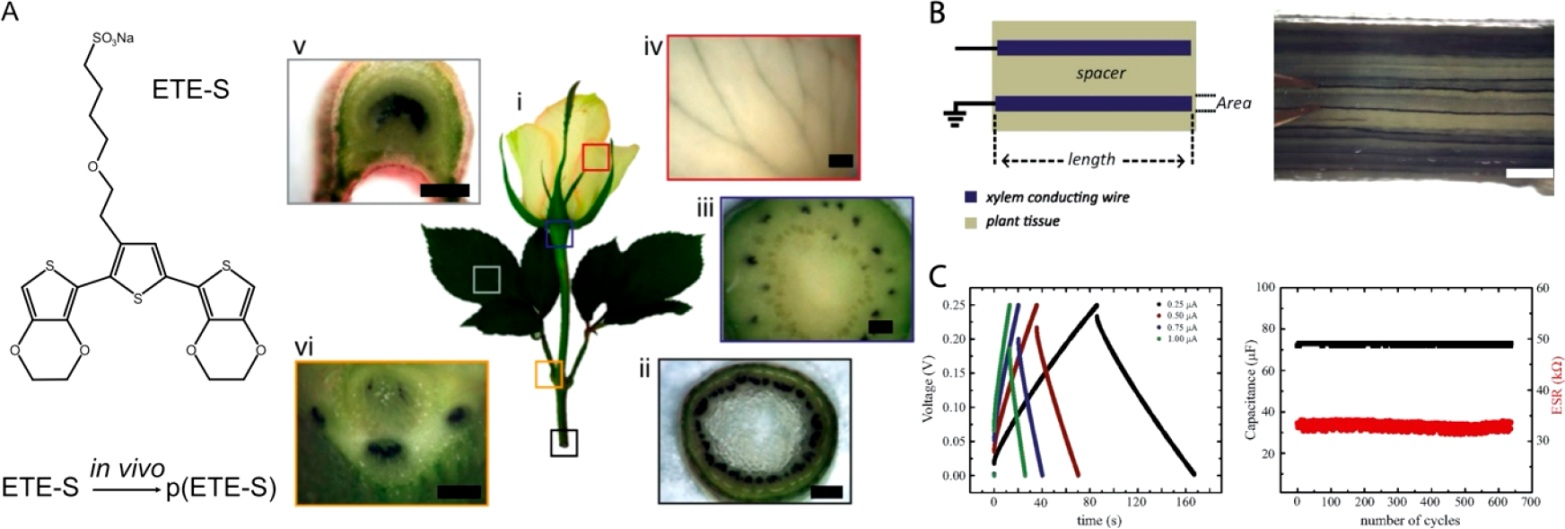
ETE-S in vivo polymerization in a rose cutting and plant supercapacitor. (A) Optical micrographs of rose (i), stem (ii, iii), petiole (vi), and leaf (v) cross sections and of the vascular bundles and rose of a rose after 24 h of immersion in an aqueous ETE-S solution. Vascular bundles darkened due to polymer deposition. (B) Schematic and micrograph of the modified xylem supercapacitor. (C) Characterization of the p(ETE-S) xylem supercapacitor with charge/discharge curves for different applied currents and stability measurement over 500 cycles. Scale bars for 1 mm. Reprinted with permission from ref (117). Copyright 2017 United States National Academy of Sciences.

In order to extend the functionalization of the plant, we developed in a following work a conjugated oligomer, the sodium salt of bis(3,4-ethylenedioxythiophene)-3-thiophene butyric acid (ETE-S).^117^ Because of its oligomeric nature, the ETE-S was transported throughout the vascular tissue of the plant from the stem to the leaves and flower while PEDOT-S was localized only in the vascular tissue of the stem. Furthermore, ETE-S polymerized in vivo with the plant acting as catalyst and template for the polymerization reaction (Figure 13A). UV–vis absorption and emission spectroscopy of polymer-xylem extracts in combination with density functional theory (DFT) calculations suggested that ETE-S was indeed polymerized, forming chains of four or more trimers. The conductivity of ETE-S was found to be 2 orders of magnitude higher than the previously characterized PEDOT-S:H wires in plants, reaching 10 S cm^–1^. Molecular dynamics revealed the formation of crystallites when ETE-S oligomers are let to self-organize.^118^ The long conducting wires and the natural architecture of the plant allowed us to fabricate a xylem supercapacitor that was stable over cycling, showing excellent charge retention and Coulombic efficiency over 500 cycles (Figure 13B,C). We stored up to 0.25 mF while more charge can be stored if more wires are connected together. ETE-S polymerization using the plant’s physicochemical environment brings new paradigms for integrating bioelectronic interfaces in tissue where materials can be phytochemically synthesized/assembled at the area of interest.

**Figure 14:**
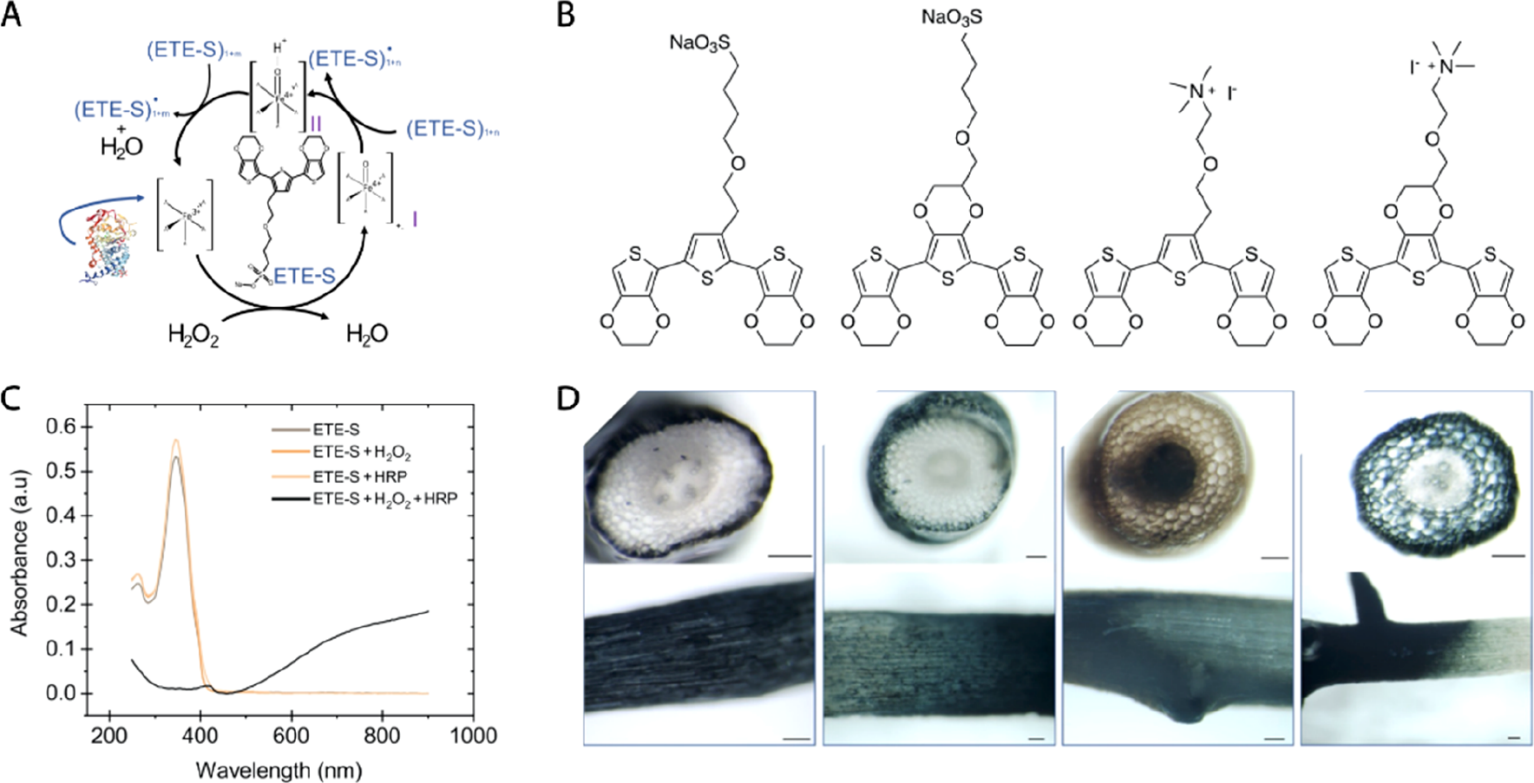
Enzymatically assisted polymerization of ETE-S. (A) Proposed mechanism of the peroxidative polymerization of ETE-S drove by the reduction of hydrogen peroxide to water. Peroxidase visuals are obtained from the NGL viewer.^121,122^ Reprinted with permission from ref (119). Copyright 2020 The Royal Society of Chemistry. Distributed under a CC BY-NC 3.0 license (http://creativecommons.org/licenses/by-nc/3.0/). (B) Schematic of the ETE-S, EEE-S, ETE-N, and EEE-N trimers. (C) UV–vis spectra of the enzymatic polymerization of ETE-S upon the addition of hydrogen peroxide and plant peroxidase (HRP). (D) Optical micrographs of root cross section (top) and top view (bottom) performed 24 h after root immersion in a 1 mM aqueous solution of trimer. Scale bar for 100 μm. Reprinted from ref (123). Copyright 2020 American Chemical Society.

At the time of the publication of the work, though, the polymerization mechanism was not clear. We speculated that ETE-S polymerized due to release of ROS such as H_2_O_2_ as they are strong oxidative species. Furthermore, ROS accumulate in the plant as a defense mechanism and ETE-S could have been perceived as an elicitor by the plant. In a later work, we elucidated the polymerization mechanism of ETE-S in the plant and discovered that ROS were involved in the polymerization but through plant peroxidases activity (Figure 14A).^119^ Plant peroxidases not only regulate the hydrogen peroxide concentration but are also involved in tuning the plant cell wall density. Depending on the conditions, peroxidases can either cross-link lipids to densify the cell wall (with lignin or suberin) or induce an oxidative burst for loosening the cell wall that can, ultimately, cause cell senescence.^120^ Via UV–vis absorption spectroscopy, we demonstrated that ETE-S polymerizes in vitro in the presence of the peroxidase enzyme and H_2_O_2_ (Figure 14C). While the enzymatic polymerization of conjugated polymers such as PEDOT and polyaniline required an acidic pH or the use of a polymerization initiator,^114^ ETE-S polymerized both in vitro and in vivo in the plant. Therefore, p(ETE-S) represents a new class of conjugated polymers that can be polymerized enzymatically in physiological conditions. With a staining method, we also confirmed that the localization of the ETE-S polymerization was correlating with the localization of the active peroxidases in plants. Our findings indicated that the ETE-S hacks a biochemical pathway in the plant, enters the peroxidative cycle of the plant cell wall and polymerizes within the cell wall structure.

In a subsequent work, we extended the molecules that can be polymerized enzymatically and in the plant by synthesizing three new oligomers, ETE-N, EEE-S, and the EEE-N, where EEE corresponds to an EDOT trimer and N represents an alkyl chain with a trimethylammonium cationic group (Figure 14B).^123^ While EEE-S was enzymatically polymerized as effectively as ETE-S, the other oligomers, ETE-N and EEE-N, polymerized in a much lower degree. Furthermore, ETE-N and EEE-N were only partially doped due to the presence of the cationic side chain that does not favor the charge stabilization on the backbone. In contrast, the trimers with the sulfone side chain, ETE-S and EEE-S, are fully doped when polymerized as the sulfonate acts as the dopant. We also demonstrated the polymerization of the trimers in vivo along the roots of bean plants. While ETE-S and EEE-S polymerized in the surface of the root, ETE-N and EEE-N entered the internal structure of the root and polymerized on the cell wall of the cortex cells (Figure 14D). ETE-N polymerized even deeper in the structure of the root and reached the plant vascular tissue. Several reasons could be responsible for the different behavior of the trimers within the plant including charge, solubility, and polymerization kinetics. Still more investigation is required to pinpoint the exact mechanism in order to enable rational design of materials for plant functionalization.

Recently, we demonstrated biohybrid plants with an electronic root system. Simply by watering the plant with ETE-S, an extended network of conductors was formed along the root system of bean plants.^124^ The p(ETE-S)-functionalized roots had conductivity in the order of 10 S cm^–1^, and their conductivity remained stable for 4 weeks even though the plant continued to grow. X-ray scattering studies revealed that the plant drives the spatial organization of the polymer resulting in enhanced π–π stacking. The functionalized roots were used as charge storage electrodes for supercapacitors that outperformed the previous demonstration of plant biohybrid supercapacitors in the xylem tissue. Finally, we investigated the development of the root system after electronic functionalization and found that the root growth and complexity is enhanced, possibly because the plant is adapting to the new hybrid state.

The work on Electronic Plants started from an exploratory basis at conceptual level of merging plants with electronics and circuits while having the structure of the plant as a template for the electronics. We demonstrated the possibility of utilizing the plant chemistry for in situ synthesis of conductors and, most importantly, the ability to directly modify with tailored material the plant cell wall that is the main component of various structural materials. Our latest findings demonstrate that electronic components can be integrated for long-term in intact plants without affecting the plant development.

### 6.2 Plant Nanobionics

Michael Strano’s group at MIT pioneered the field of Plant Nanobionics. Plants are functionalized with nanomaterials that enhance their native processes such as photosynthesis. In this case, the functional nanomaterials have an active role in the plant, going beyond delivery of macro- or micronutrients that is the role of nanofertilizers.^125,126^ Plant Nanobionics also aim to convert plants into devices, for example, environmental sensors or light emitting plants. Here the introduced nanomaterials induce a non-native functionality to the plant.

**Figure 15:**
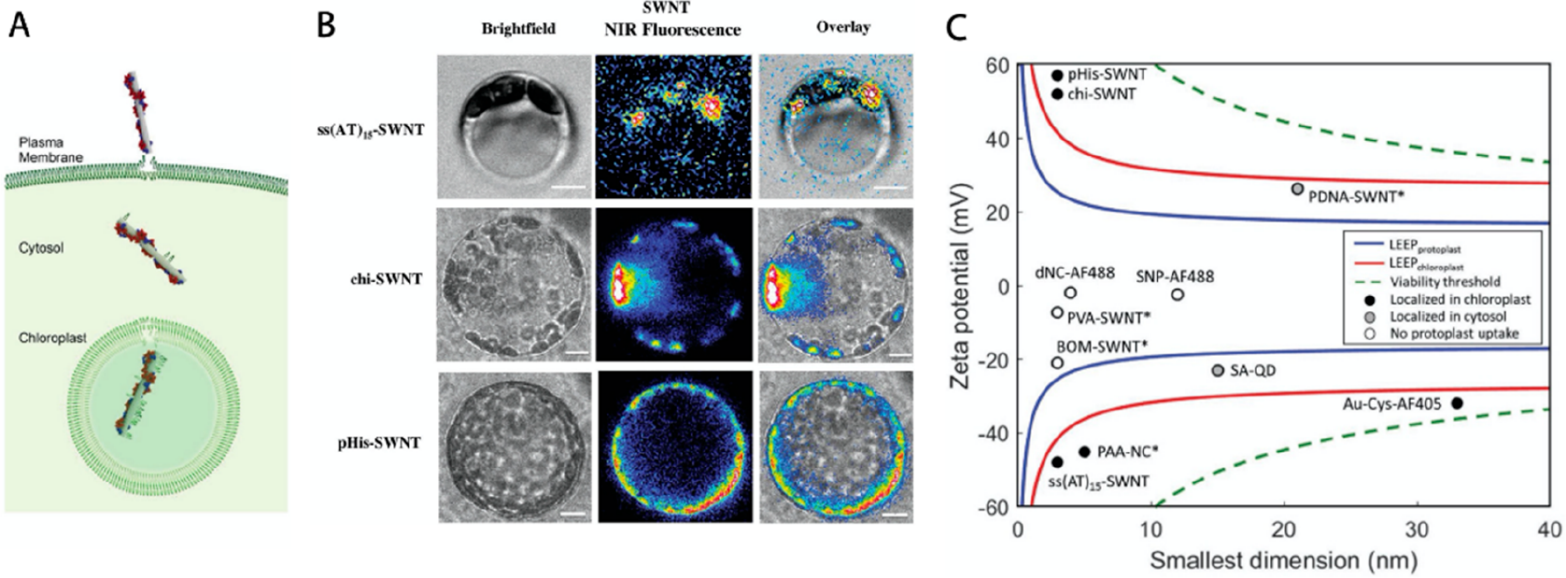
Interaction between nanoparticles and plant cell membranes. (A) Illustration of the nanoparticle behavior when nanoparticles can cross the protoplasts membrane and end up localized in the cytosol or additionally cross the chloroplasts double membrane and stay entrapped in the organelle. (B) Bright field, nIR fluorescence, and overlay images of protoplasts incubated with SWCNTs. The intensity of the surface charge, rather than its charge, follows the chloroplasts colocalization trend. Scale bar for 5 μm. (C) LEEP model for the entry of nanoparticles inside protoplasts. Blue and red lines respectively represent the LEEP model for the protoplasts and chloroplasts entry. Reprinted with permission from ref (129). Copyright 2018 John Wiley and Sons.

Plant nanobionics rely on smart nanomaterials that localize within the plant tissue, extracellularly, or even intracellularly within organelles such as chloroplasts. Nanoparticle uptake into plant cells requires the particle to pass across the plant cell wall and the cell membrane. While initial studies demonstrated uptake of functionalized single-walled carbon nanotubes (SWCNTs), the mechanism of uptake was not well understood.^127^ Wong et al. performed a systematic study with focus on localization of nanoparticles in the chloroplasts.^128^ A series of materials were tested with different sizes and ζ-potential. Interestingly, it was found that the absolute charge and not the sign of the ζ-potential was determining the spontaneous localization within the chloroplast as verified via confocal microscopy. The interaction of single-stranded DNA (ssDNA) and water-soluble modified nanotubes going across the lipid membrane was also demonstrated via solvatrochromic shift and fluorescence quenching in the nIR during the chloroplasts’ internalization process. The authors developed a mathematical model called the lipid exchange envelope and penetration mechanism (LEEP) that describes the penetration of nanomaterials through the chloroplast’s envelope (membrane) (Figure 15A). According to the LEEP, highly charged particles would induce a transmembrane potential that causes a pore formation and enables the transport of the nanoparticle within the organelle (Figure 15B). The radius of the induced pore should be below a certain threshold to prevent lysis of the cell or organelle. In order to describe whether a nanoparticle will be localized in the cytosol or within the chloroplast, Lew et al. extended the LEEP model taking into account the dielectric constant of the membrane, as it determines the transmembrane potential and consequently the pore formation (Figure 15C).^129^ The lipidic composition of the cell membrane is different from the one of the chloroplast envelope that also has a double membrane. The cell membrane has a higher dielectric constant than that of the chloroplast, and thus, nanoparticles with lower charge induce a sufficient transmembrane potential to enter the cytosol but not sufficient to enter the chloroplast. For chloroplast localization, nanoparticles with higher ζ-potential are required.

**Figure 16:**
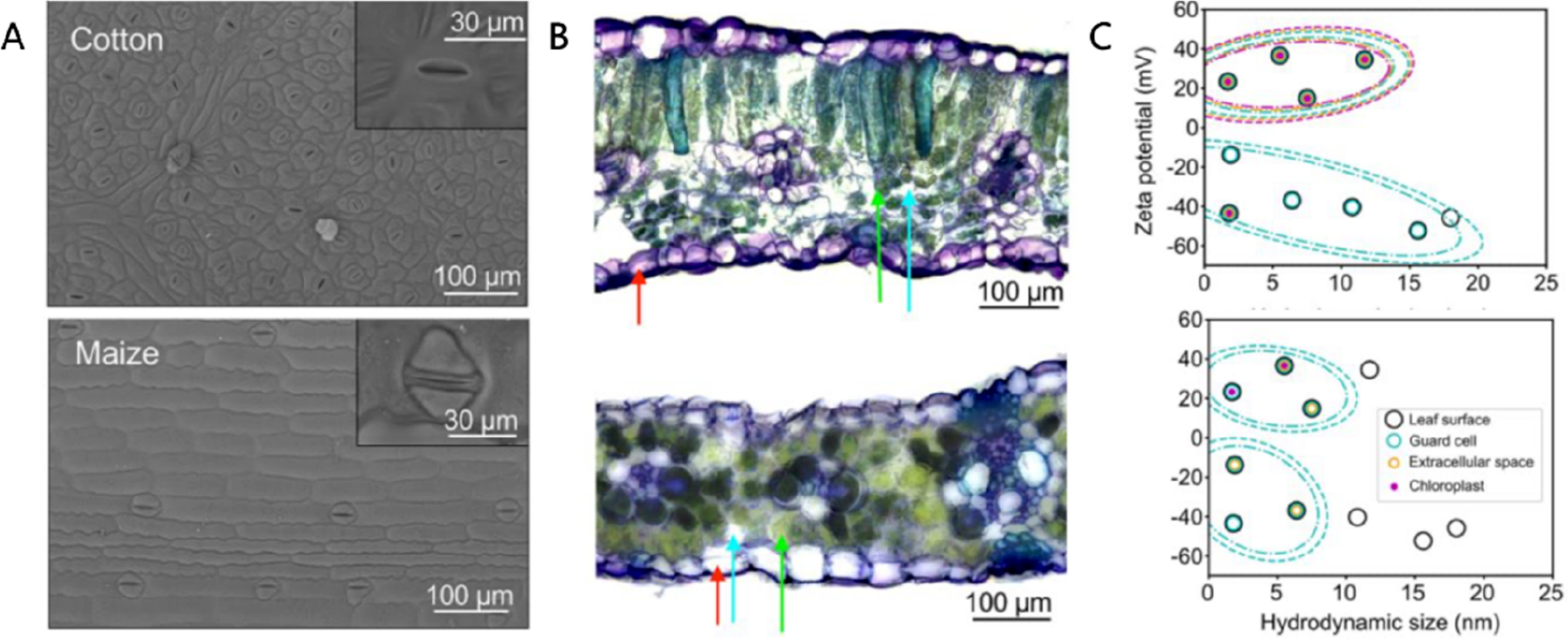
Leaf anatomical importance toward nanoparticle internalization. (A) SEM images of cotton and maize leaf surface showing the structural difference of stomatal density and arrangement from plant to plant. (B) Bright field of maize and cotton leaves cross sections showing difference in their anatomy. Red arrows point to guard cells while blue arrow point to extracellular space and green arrow point to chloroplasts. (C) Nanoparticle leaf empirical model for maize and cotton predicting the size and zeta potential required to internalized nanomaterial in the guard cell. Reprinted with permission from ref (130). Copyright 2020 American Chemical Society.

While these studies reveal the mechanism involved in the internalization of nanoparticles by plant cells, they do not take into account the leaf cuticle or mesophyll. Foliar delivery of nanoparticles faces physical barriers that will depend on the plant species (Figure 16A,B).^130^ Hu et al. showed that nanoparticles up to 18 nm can be uptaken by cotton leaves, while maize leaves were impermeable to particles with a hydrodynamic diameter bigger than 8 nm (Figure 16C). Confocal microscopy of both the epidermis and the mesophyll compartments during the foliar delivery underlined that nanoparticle uptake was following both stomatal and cuticular pathways for the dicot plant (cotton), while in the monocot plant (maize) uptake was mostly through the stomatal pores. In the next section we will overview some applications of plant nanobionics.

### 6.3 Plant Nanobionics Applications

#### 6.3.1 Nanomaterials for Augmenting Plant Functions

The first Plant Nanobionics application focused on improving the photosynthetic yield of plants by introducing functionalized carbon nanotubes into the photosynthetic organelles, the chloroplasts. This work preceded the LEEP model; therefore, at that time, it was not clear what defined the localization of nanomaterials within the chloroplasts. Functionalized SWCNTs were infiltrated into spinach (*Spinacia oleracea*) leaves and enhanced the photosynthetic activity of the plant as it was indicated by improved electron transport rates. With in vitro experiments using extracted chloroplasts, the authors showed that SWCNTs increased the reduction rate of 2,6-dichlorophenolindophenol (DCPIP), an electron mediator between the photosystem II and photosystem I, confirming that the nanotubes were facilitating the electron transport. Furthermore, SWCNTs, due to their semiconducting nature, could act as antennas extending the utilizable light spectra of photosynthesis. The hypothesis was corroborated by in vitro experiments where metallic carbon nanotubes did not enhance the photosynthetic efficiency of isolated chloroplasts.

Wu et al. investigated the effect of cerium nanoparticles (nanoceria) on plant photosynthesis under stress conditions. Nanocerias were chosen because of their ability to catalytically quench stress-induced ROS.^131^ ROS can cause irreversible damage to chloroplasts and therefore greatly threaten plant survival. Plants treated with negatively charged nanoceria particles that scavenge both superoxide anion and hydrogen peroxide were more tolerant to high illumination stress in comparison with control plants as well as plants treated with positively charged nanoceria particles or negative particles specific to superoxide anion only. Additionally, plants with internalized negatively charged nanoceria particles had more tolerance to high or low temperature as carbon assimilation or RuBisCO activity were less impacted than untreated plants. The authors also correlated the uptake of nanoceria and localization within the chloroplasts with their charge. In contrast with the LEEP model, in this case the charge sign and not its magnitude defined the localization. The negatively charged nanoceria showed two times higher chloroplast localization than the positively charged ones. The authors hypothesize that this is because of the interaction of the negatively charged nanoceria with the positively charged plant cell membrane. By inducing membrane depolarization with incubation in NaCl, fewer positively charged nanoceria were uptaken by the cell, while the uptake of the negatively charged nanoceria increased, confirming this hypothesis. Moreover, it was suggested that nanoparticles follow a nonendocytosis pathway as the uptake was not temperature dependent.

Carbon dots (CDs) have also received attention in plant applications. During the past years, CDs have been widely studied due to their easy and affordable synthesis in combination with their remarkable fluorescence properties that can be tuned during synthesis.^132^ Because of their size, generally in the tens of nanometer range, these materials can be easily infused in the plant to image roots, stems, and leaves, and in some cases, it was reported that they were internalized in the plant cells. Few articles report the positive impact of carbon dot assimilation on plant growth.^133−135^ The beneficial effect of CDs on plants was attributed to various factors including the increase of seed wettability that promotes their germination^136,137^ but also to intracellular phenomena where CDs interaction with DNA resulted in modification of gene expression in rice plants.^134^ The optoelectronic properties of CDs were also leveraged to enhance the photosynthetic rate.^138^ Although many studies have been published, still many questions remain unanswered on the effect of CDs on plant physiology. CDs have also been widely used as nanosensors due to modulation of their fluorescence properties in the presence of various analytes.^139^ Thus, there is potential for applying these materials as a sensing platform in living plants. Readers are referred to more detailed reviews on CDs focusing on synthesis,^132^ optical properties, and reported effects on plants.^140^

Conjugated polymer nanoparticles were also used as light transduction units for plant physiology modulation. Recently, the Antognazza group demonstrated the use of poly(3-hexylthiophene (P3HT) nanoparticles for light-induced modulation of stomata aperture.^141^ Leaf epidermal strips of *Arabidopsis thaliana* were incubated in a P3HT nanoparticles solution. The nanoparticles were not internalized by the leaf tissue or entered the cytosol of guard cells but remained in the extracellular bath. However, when the epidermal strip was illuminated with white light for 90 min, the stomatal aperture decreased significantly for the P3HT-treated samples in comparison with untreated samples or samples incubated with optically inert silica nanoparticles of similar size. The authors hypothesized that P3HT is acting as an oxygen photocathode that generated ROS that would trigger the stomata closure. Additionally, they demonstrated that P3HT beads affected the oscillations of cytosolic calcium concentration, specifically under a green light stimulation that corresponds to the absorbance peak of P3HT (λ = 540 nm). The photoexcitation of the polymer reduced significantly both the cytosolic Ca^2+^ oscillations number and amplitude. Ca^2+^ modulation has been proposed as a direct intermediary in the stomata closure mechanism, and therefore, this example shows that P3HT nanoparticles are good candidates for optical regulation of Ca^2+^ concentrations and stomatal modulation. Using the optoelectronic properties of conjugated polymer nanoparticles for controlling stomata function on demand with light can be used to tune plant’s water consumption, for example, in drought conditions.

#### 6.3.2 Nanosensors for In Vivo Monitoring of Plant Physiology

Functionalized carbon nanotubes can be converted to sensors for in vivo monitoring of analytes via corona phase molecular recognition (CoPhMoRe).^142^ This method uses the macromolecular assembly of amphiphilic polymers on carbon nanotubes to create a selective molecular recognition site for a specific analyte. When the analyte binds to the modified nanotube, it will induce a modulation of its fluorescence signal, either a wavelength shift or an intensity modulation, that can then be used as a readout. In many cases, ssDNA is used for the nanotube modification due to the large number of nucleotide combinations that can bring a specific binding for one analyte. Giraldo et al. demonstrated the possibility to use CoPhMoRe sensors in plant biosensing with DNA-functionalized SWCNT for the detection of dissolved nitric oxide in extracted chloroplasts and spinach leaves.^143^ The specificity and reversibility of this sensor showed that endogenous molecules sensing can be done with a high spatiotemporal resolution.

A few years later, Lew et al. developed a nanosensor for in vivo detection of H_2_O_2_, which is a long-distance signaling molecule in plants related to defense responses.^144^ The DNA-wrapped SWCNT nanosensors could be used for sensing physiological concentrations of H_2_O_2_ as their fluorescent intensity was quenched with a high selectivity and a dynamic range from micromolar to millimolar in vitro and in vivo. The sensors’ response was reversible in vitro in the presence of catalase that converts H_2_O_2_ in H_2_O and O_2,_ suggesting that the sensors can be regenerated in vivo as well from native enzymes and therefore can be applied for long-term monitoring. H_2_O_2_ nanosensors and control nanotubes were infiltrated into spinach leaves and localized in the plasma membrane and in the chloroplasts. nIR fluorescence was monitored at a standoff distance of 1 m via a 2D array InGaAs detector. When wounding was induced on the leaf surface, the nanosensor fluorescence initially decreased rapidly and then recovered within 10–20 min, while the fluorescence of the control nanotubes remained unchanged. The recovery phase is attributed to H_2_O_2_ unbinding or its decomposition by antioxidants or enzymes. The waveform of the sensors’ fluorescent response was found to be dependent on the type of stress that was inducing the H_2_O_2_ signal; therefore, it can be used to differentiate various stresses such as high heat, light, and wounding. The signal propagation could be monitored with a high spatiotemporal resolution, and it was found that the H_2_O_2_ wave travels faster in the vasculature, with a speed similar to the wound-induced electrical and Ca^2+^ signals. This finding supports the hypothesis that H_2_O_2_, electrical signals, and Ca^2+^ are all interrelated within the plants defense responses. The H_2_O_2_ nanosensors were effective in a variety of plant species such as spinach, strawberry blite (*Blitum capitatum*), lettuce (*Lactuca sativa*), *Arabidopsis*, sorrel (*Rumex acetosa*), and arugula (*Eruca vesicaria*), although the signal waveform varied between species, suggesting that the defense mechanism of plants has evolved based on their native environment. Furthermore, the applicability of the method in various species highlights the potential of the nanobionics technology for elucidating plant signaling without the need of genetically encoded sensors that are usually developed in model species. Detection of the signal using low-cost electronics was also possible, opening the pathway for field application, for example, for early detection of abiotic and biotic stress.

Nanoprobes based on quantum dots (QDs) were also demonstrated by Wu et al. for monitoring glucose.^145^ In order to obtain glucose specificity, QDs were modified with a tetraphenylene fluorescent probe with a boronic acid pendant group that undergoes esterification in the presence of glucose, thus quenching the initial fluorescence of the probe.^146^ The fluorescence quenching was found to be dependent on glucose concentration with a linear response between 100 and 1000 μM. Although, in principle, the method can have single chloroplast resolution, no detection of endogenous glucose exported from chloroplasts was achieved. Detection was only possible after infiltration of a 500 μM glucose solution into *Arabidopsis thaliana* leaves and in algae (*Chara zeylanica*). Therefore, these probes will require more optimization to show a real application in plant systems.

#### 6.3.3 Plant-Based Environmental Sensors

**Figure 17:**
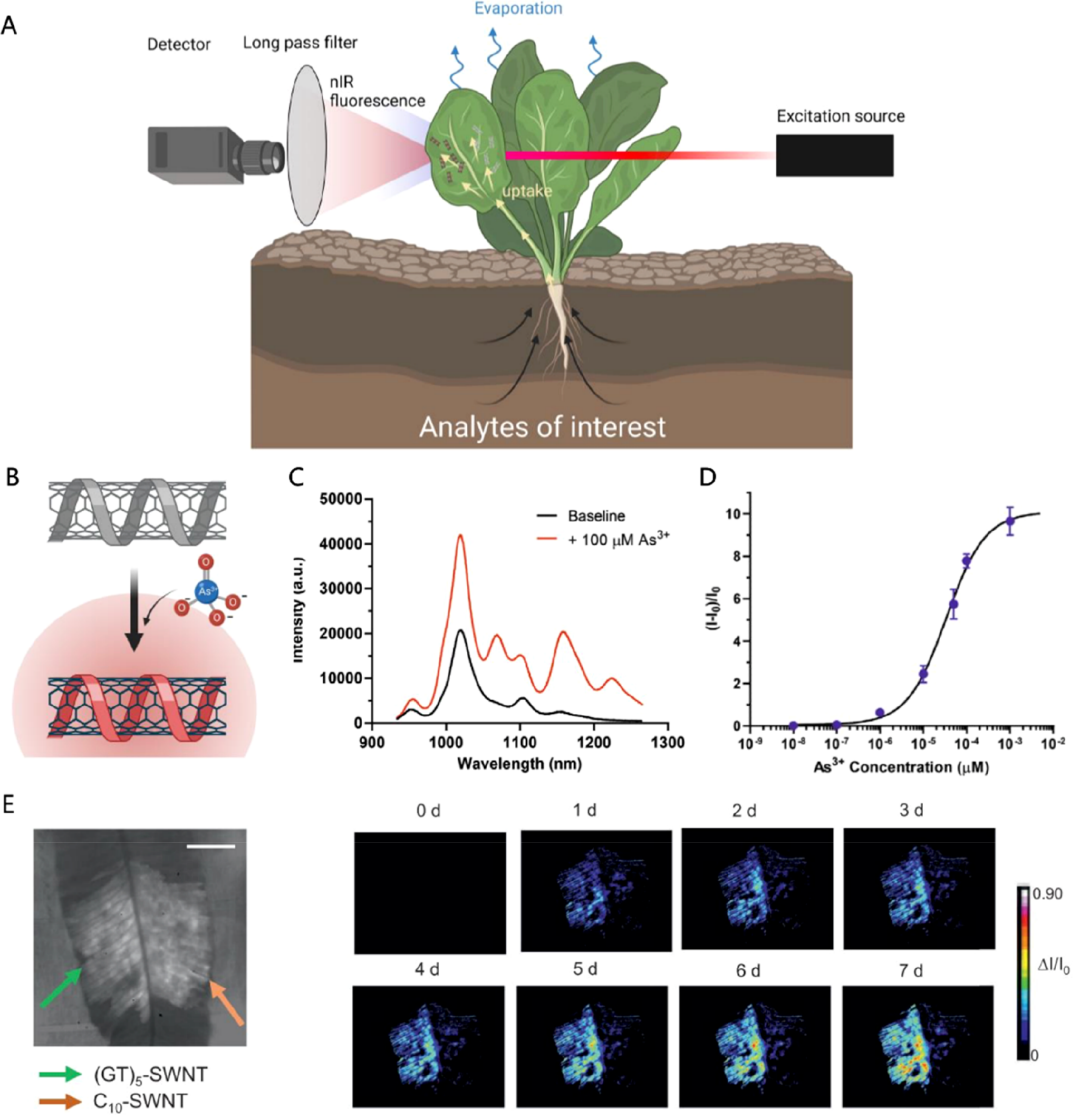
(A) Schematic of the standoff environmental detection using the microfluidics vasculatures of intact plants to bring analytes from the soil to the leaves, modified with nanobionic sensors. (B) Schematic of the induced fluorescence modulation of arsenite on the SWCNTs. (C) nIR Fluorescence trace of the (GT)_5_-SWCNT before and after the addition of 100 μM As^3+^. (D) Calibration curve of the arsenite sensor where arsenite concentration is plotted versus the normalized response of the sensor (*I*_0_ – *I*)/*I*_0_. (E) Bright field image of a *Pteris cretica* leaf infiltrated with (GT)_5_-SWCNT (green arrow) and (orange arrow). The increase of the nIR fluorescence is observable only on the leaf side infiltrated with carbon nanotubes specific for arsenite detection. Adapted with permission from ref (149). Copyright 2020 John Wiley and Sons.

Using the same principle for in vivo endogenous molecule detection via nanoparticles, a plant can be converted into an environmental sensor for detection of exogenous analytes. Through the process of transpiration, water and nutrients travel from the soil to the leaves. Therefore, the plant can act as a sampler of the soil but also an analyte collector (Figure 17A). The first demonstration of a plant-based environmental sensor was developed by Giraldo et al. for the detection of nitroaromatic compounds that are a class of pollutants found in explosives.^147,148^ SWCNTs were functionalized with a peptide from the bombolitin family (B-SWCNT), which, upon interaction with picric acid, expresses a quenched fluorescence in the nIR. B-SWCNT and control nanotubes functionalized with poly(vinyl alcohol) (PVA) were infiltrated into the plant leaves in two different locations. After 5–15 min of plant exposure to a picric acid solution, the B-SWCNT fluorescence decreased while the PVA-SWCNT signal was unaffected. The same sensing abilities were shown when the picric acid was directly applied on the leaf. The change of the fluorescence was detected with a high-end IR camera based on InGaAs detectors. In order to prove the applicability of the sensor in field conditions, the authors also demonstrated the potential of a stand-alone plant sensor with the use of a portable low-cost detector based on a Raspberry Pi CCD detector, allowing the real time wireless transmission of images to a smart phone.

This work showed that the architecture of the plant provides a robust network of living microfluidics engineered through evolution that can be used for environmental sensing. However, this approach is limited by the accumulation of the analytes in the leaves that can lead to sensor saturation. Even though transpiration is a process shared by all plants, hyperaccumulator plants have developed resilience to toxic compounds found in the soil. One example is *Pteris cretica*, which is a hyperaccumulator of arsenic.^149^ Lew et al. developed a plant sensor for the detection of arsenite, the most common form of arsenic present in paddy soil, called (GT)_5_-SWCNT. SWCNTs were functionalized with a guanine/thymine polymer as these nucleobases create strong hydrogen bonds with the hydroxyl groups of arsenite.^150^ After 30 min of immersing a spinach plant into the arsenite solution, the sensor fluorescence was significantly higher than the control signal, reaching a 11% of fluorescence increase after 5 h (Figure 17B–D). A similar detection was obtained with rice crops and resulted in 15% increase after 5 h, showing that this sensor could detect traces of arsenite pollutant in widely used crops. These common plants were then compared with the hyperaccumulator *Pteris cretica.* After 1 week, the amount of arsenite detected in *Pteris cretica* was 74% higher than in the spinach and rice samples, demonstrating the tolerance of *Pteris cretica* toward arsenite for sensing and the possibility of using this plant for depolluting soils (Figure 17E).

#### 6.3.4 Light-Emitting Plant

**Figure 18:**
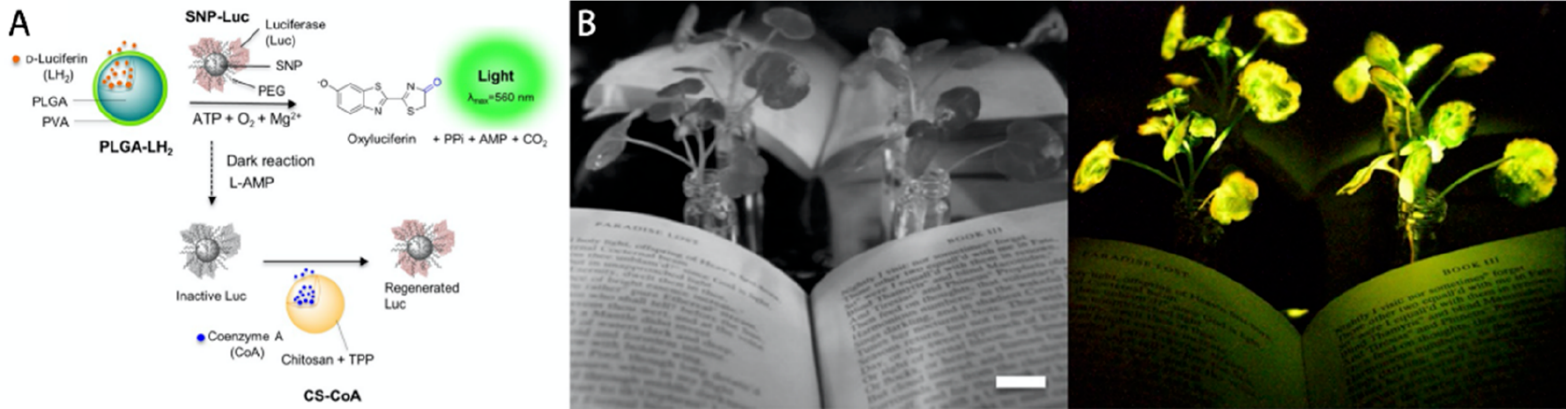
Nanobionic light emitting plant. (A) Reaction mechanism of the Firefly luciferase in the presence of luciferin and plant physiological ATP, the coenzyme A is used to regenerate the luciferase after reaction. Luciferin, the luciferase, and the coenzyme A are represented with their corresponding nanoparticle carriers. (B) Book illumination using a modified watercress as a light emitting plant. Reprinted (adapted) from ref (154). Copyright 2017 American Chemical Society.

Plant-based devices can be seamlessly integrated in the urban environment combining the natural comfort of plants with device functionalities serving the city, for example, converting trees into streetlights. Some species, such as firefly, algae, or fungi, express bioluminescence, an ability that plants do not naturally have. Light-emitting transgenic tobacco plants have been reported via introduction of bioluminescence genes from firefly,^151^ bacteria,^152^ or fungi.^153^ When the plant was modified to express the firefly luciferase, an external luciferin source was required for light emission,^151^ while when the bacteria luciferase pathway was introduced, light emission did not require any external supply of substrate (autoluminescent).^152^ Recently, a transgenic plant with integrated fungal caffeic acid cycle was reported with enhanced luminescence.^153^ The concept was further explored by Kwak et al. that developed a nanobionic light emitting plant based on the chemiluminescence of luciferin without the need of genetic modification, powered by ATP from the plant mesophyll cells (Figure 18).^154^ Silica nanoparticles coupled with luciferase enzymes (SNP-Luc) were designed to access the mesophyll and guard cells to reach the location of ATP sources. Nanoparticles carrying d-luciferin and others carrying the coenzyme A, the reactant and enzyme regenerator, respectively, were designed to remain in the mesophylls’ extracellular space where they will unload their reagents. The nanoparticles were infiltrated using a pressurized chamber that enables the infiltration of intact 1 month old watercress, arugula, and spinach. It was found that the incubation of SNP-Luc before the injection of the other reactive nanoparticles was promoting the diffusion of the luciferase particles inside the guard cells, where the concentration of ATP reaches 1 mM (3 orders of magnitude higher than in the extracellular space), therefore increasing the intensity as well as the duration of the light emission. Preinfiltration of mesoporous silica nanoparticles also extended the light emission duration for 3 h by slowing the diffusion of the reagents inside the leaf mesophylls. Designing SNP-Luc with a higher ζ-potential could also help to reach higher intensity by aiding its diffusion across the cell membranes and therefore reaching higher concentration of ATP. Another example of light emitting plants was recently reported based on phosphorescent particles that were infiltrated in the leaves of different plants species found commercially, including one tree. The particles were based on strontium aluminate (SrAl2O4:Eu^2+^,Dy^3+^) that was milled to form particles with a size of hundred nanometers range and then coated with SiO_2_ to reduce its phytotoxicity. The particles were excited with a 400 nm LED for 10 s resulting in phosphorescent emission for a period of 5 min. The authors demonstrated that the particles could be charged and discharged within the plant for 16 h a day (2016 cycles) for 2 weeks without any effect on plant physiology.^155^

Plant nanobionics incorporate nanomaterials in plants for developing versatile technological solutions such as environmental sensing or plant physiology monitoring. Via chemical modification, nanoparticles can be converted to nanosensors for monitoring endogenous or exogenous molecules with high spatiotemporal resolution. Bioelectronic devices, on the other hand, are more invasive due to larger footprint and can only monitor locally at the site of insertion. However, more studies are needed to understand the effect of wide distribution of nanomaterials on plant signaling as they may interfere with natural processes. Furthermore, utilizing the optoelectronic properties of nanomaterials can be a handle for light stimulation of plant signaling as an alternative to optogenetics.

## 7 Energy Harvesting from Living Plants

Every day, we harvest energy from plants either in the form of food or via combustion of fossil fuels and biofuels. Harvesting energy from plants without compromising their growth is also possible via biofuel cells and triboelectric generators. The power output of these devices though is low; therefore, they can be used as energy sources of niche applications. For example, the emerging area of Internet-of-Things imposes the need to develop low-cost and environmentally friendly methods to power a vast number of widely distributed sensors or smart devices of low power consumption. These devices could be related to plant monitoring, for example, in smart agriculture, or can take advantage of the widespread location of plants in remote areas to enable development of self-powered energy stations.

### 7.1 Biofuel Cells

Enzymatic biofuel cells are devices that convert chemical energy into electricity via oxidoreductase enzymes that catalyze the reactions at an anode and a cathode. The most widely explored biofuel cells for bioelectronic applications are based on glucose oxidation and oxygen reduction as both glucose and oxygen are present in high concentrations in bodily fluids. So far, most of the focus on implanted biofuel cells has been placed toward the field of medicine because of the potential to continuously power implanted devices such as pacemakers, glucose sensors, or brain probes.^156^

**Figure 19:**
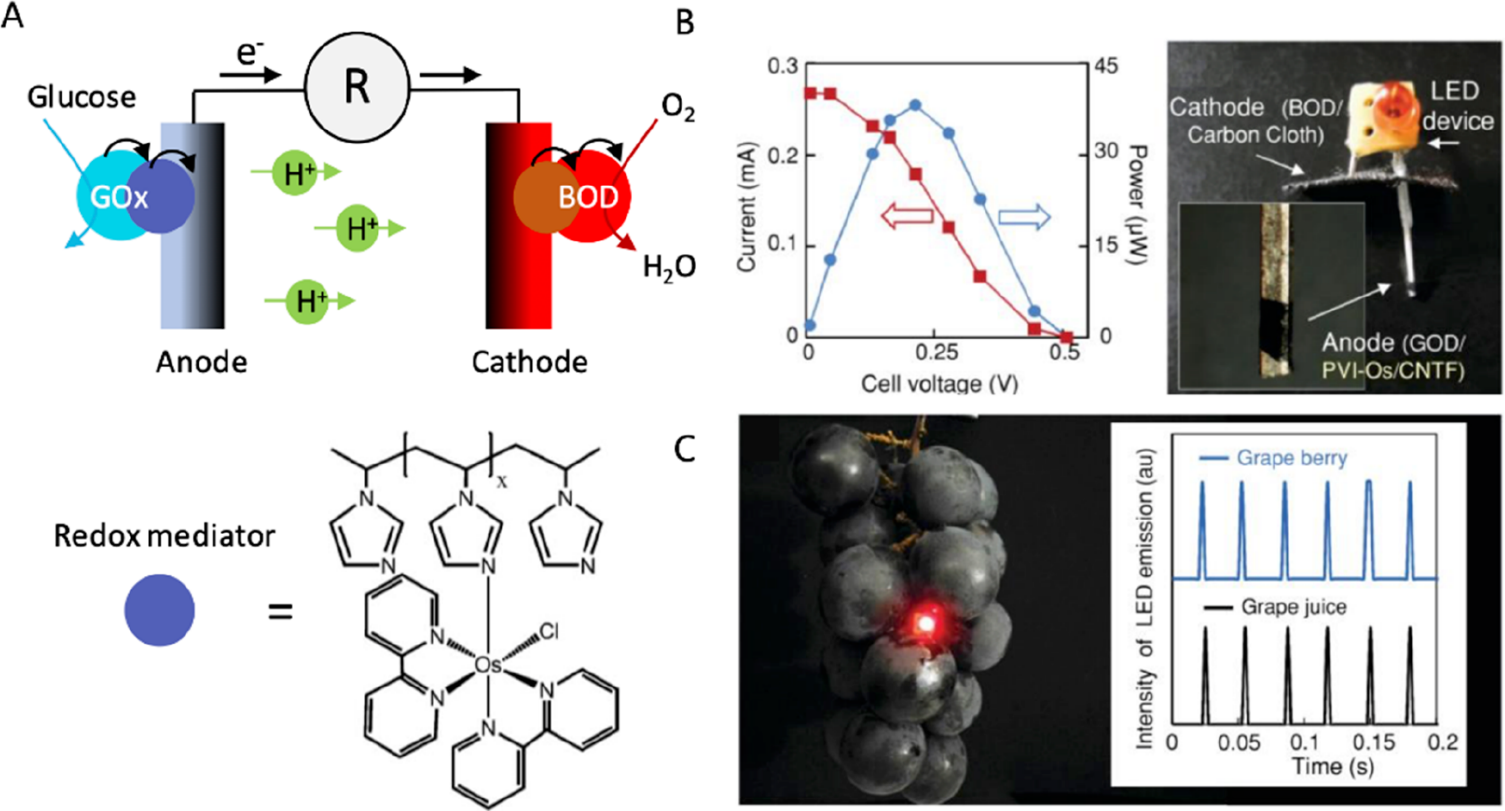
(A) Schematic of an enzymatic biofuel cell using glucose oxidase as an oxidative enzyme for the anode and bilirubin oxidase as a reducing enzyme at the cathode. Example of an osmium mediator (poly(vinyl imidazole)-bis(2,2-bypiridine-*N*,*N*′) Os^II^) used to shuttle electron between the enzyme and the electrode. (B) Performance and picture of a needle-based biofuel cell in 200 mM glucose. The device is obtained by using a carbon cloth with bilirubin oxidase as an oxygen diffusion cathode and a carbon nanotubes forest (CNTF) modified with glucose oxidase and a mediator as an anode. (C) Application of the needle biofuel cell that powers a red LED, blinking in the presence of glucose. Adapted with permission from ref (160). Copyright 2012 John Wiley and Sons.

The first biofuel cell in plants was demonstrated in a grape by Mano et al. based on carbon fiber electrodes functionalized with osmium redox polymers, glucose oxidase, and bilirubin oxidase enzymes (Figure 19A).^157^ Redox polymers mediate the electron transfer between electrodes and enzymes and offer a matrix for enzyme immobilization. Furthermore, redox polymers enable the development of membrane-free biofuel cells that are more versatile and can be therefore easily implanted in living systems. The grape-biofuel cell operated at a potential of 0.52 V and retained 78% of its initial power after being inserted for 1 day in the fruit. A similar system was later demonstrated in a cactus plant.^158^ In that case, the electrodes were based on carbon rods and were implanted into the cactus ground tissue. The power output of the biofuel cell under light increased by 70% compared to the dark conditions; thus, the authors speculated that this was a result of plant photosynthesis that increased the glucose and oxygen concentration. However, the anodic and cathodic currents changed only few seconds after illumination, which would suggest that the electrodes were in direct contact with the photosynthetic machinery. As photosynthesis takes place within the chloroplasts, this seems highly improbable since the macroscopic electrodes were implanted into the ground tissue. While the power output of the grape implanted biofuel cell reaches 240 μW cm^–2^, the power generated by the cactus biofuel cell was 20 times lower (9 μW cm^–2^ under light conditions). This large difference reported for similar electrochemical systems can be explained by the difference in the amount of glucose that is found in a grape and a cactus, >20 and ∼20 μM, respectively.

A more versatile biofuel cell had a needle shape to facilitate its insertion in different living systems, including fruits.^159^ To avoid oxygen limitations at the cathode without perturbing the anode operation, the cathode was based on a gas diffusion electrode that was separated from the needle-shaped anode. The cathode was modified to be hydrophobic using carbon paper with a conductive ketjen black ink and poly(tetrafluoroethylene). The cathode was placed above the anodic needle, with its outer layer exposed to air, therefore enabling better oxygen diffusion and avoiding limitations due to the low concentration of dissolved oxygen in the host body. The needle-based grape biofuel cell had a power output of 111 μW cm^–2^, and it was used to power a small LED from which the blinking frequency increased with the fructose concentration (Figure 19C).

One way to increase the performance of biofuel cells is to enhance the electron transfer between the enzyme reaction center and the electrode. Using carbon nanotube-patterned structures called a carbon nanotube forest (CNTF), Yoshino et al. demonstrated a grape-implanted biofuel cell (Figure 19B).^160^ In this case, the structure of the CNTF enabled the adsorption of both the glucose oxidase and the poly(vinyl imidazole) (PVI)–osmium mediator in a configuration that resulted in a high biocatalytic activity. The charge transfer at the anode had a high turnover rate similar to glucose oxidase activity in the presence of oxygen.^161^ The power density of the implanted biofuel cell in vivo was measured at 3.375 mW cm^–2^, showing that the structure of the electrode can play a major role in increasing the performance of biofuel cells.

A more recent example of implanted biofuel cells in plants had an increased power output with the use of large electrodes (3.75 cm^2^) implanted in orange.^155^ The anode electrodes were engineered for non-mediated electron transport by using pyrroloquinoline-quinone (PQQ)-dependent glucose dehydrogenase and flavin adenine dinucleotide (FAD)-dependent fructose dehydrogenase that are also oxygen-independent enzymes. The anode could therefore harvest energy from two different sugars sources found in oranges, glucose and fructose, without relying on the dissolved oxygen concentration. The biofuel cell had power output of 90 μW cm^–2^ and was used to power a transmitter that sends an e-mail when the required sugar amount is converted into electricity. This operation could only be accomplished when a voltage of 2.3 V was reached, which requires the energy harvesting devices to be coupled to a circuit with two capacitors, one integrated with the energy harvesting unit (1 mF) and an external one (6.8 mF) that could be controlled to automate the transmission of information. Even though this method is destructive for the fruit, such a device could be integrated in selected fruits to act as indicators for the rest of the harvest, assuming that the device is not significantly disturbing the fruit development and may therefore give false indications.

Engineering implanted biofuel cells for long-term powering of devices is challenging for many reasons. First, enzymes lose their activity in living systems. Even if some publications reported stability over several weeks in a living snail,^162^ the enzymatic activity decreased with time, signifying that the power output cannot be stable over extended periods of times. Second, in order to reach high power, the cascade reaction leading to an electron transfer from the fuel to the electrode needs to minimize any electron losses by external factors, such as molecular oxygen for the anodic reaction. Finally, an implanted biofuel cell requires a design that will be minimally invasive to the host tissue, for example, via miniaturization and use of materials and architectures that are more compatible with the specific biological milieu.

Conjugated polymers can bring advantages in the field of “in-planted” biofuel cells because of their high surface area that can result in increased current output and their soft interface with biological milieu, extending their lifetime. Recently, Ohayon et al. demonstrated that an n-type conjugated polymer named P-90 performed as a 3-in-1 material for enzymatic biofuel cells.^163^ Glucose oxidase was physically adsorbed on the polymer without the need to use a cross-linker or covalent bonding while the polymer n-type conduction enabled direct electron transfer from the enzymatic reaction without the need of a mediator. The biofuel cell showed a performance of 2.8 μW cm^–2^ with 10 mM of glucose with an open circuit voltage (OCV) measured at 0.310 V in vitro. A maximum power of ∼23 μW cm^–2^ could be achieved when the P-90 was electrochemically doped prior to the biofuel cell operation.

The optoelectronic properties of conjugated polymers can also be explored in plant-based energy harvesting systems. The electrical and optical properties of conjugated polymers are attractive for direct interface with thylakoid membranes, the center of light conversion reactions within the chloroplasts. A conjugated polyelectrolyte called poly(9,9-bis(6′-*N*,*N*,*N*-trimethylammonium)hexyl)fluorene-*co*-*alt*-1,4-phenylene) bromide (PFP) was used to broaden the absorbance of the photosystem II, enabling the collection of light in the UV area (λ = 380 nm).^164^ Upon light illumination, an increase of the thylakoid membrane fluorescence (λ = 680 nm) was observed through Förster resonance energy transfer (FRET), while PFP fluorescence (λ = 425 nm) was quenched, indicating a complete energy transfer from the PFP to the thylakoid membrane. This biohybrid system was then electrically and electrochemically assessed on bare carbon paper and showed a four-time increase of photocurrent (1245.7 ± 41.1 nA cm^–2^ for PFP/thylakoids vs 316.6 ± 14.0 nA cm^–2^ for thylakoid membranes only) and a two-time increase of water oxidation. In a following work, PFP/thylakoids composite was used as the anode of a bioelectrochemical cell.^165^ It was found that the photocurrent collection can be greatly improved by optimizing the structure of the conjugated polyelectrolyte with different end groups or side chains that enhance the interaction with the photosynthetic apparatus.^166^

### 7.2 Triboelectric Nanogenerators

Triboelectric nanogenerators (TENG) represent an intriguing solution for harvesting otherwise wasted random and low frequency environmental mechanical energy such as ambient mechanical motion.^167,168^ TENG operation relies on the triboelectric effect (contact electrification) coupled with electrostatic induction. The simplest TENG consists of two electrodes that are coated with different dielectric materials. In short-circuit mode, when the two dielectrics are brought in transient contact, they develop opposite charges on their surface forming an electric double layer. When they are separated, mirror charges are induced at the back electrodes that can then be collected via an external circuit. Plant leaves are attractive natural materials for TENG as they are abundant, widely distributed in our surroundings, carbon negative, and move in response to ambient forces such as wind and rain. Upon friction, leaves become electrostatically charged due to the presence of a lipid crystal layer on their surface. A majority of leaves become positively charged, while leaves of only a few species can acquire negative charges, e.g., *Bryophyllum pinnatum* (known also as *Kalanchoe pinnata*).^169^ The native micro-/nanostructured leaf surface furthermore increases the effective contact area with the dielectric material resulting in an increase of the electric double-layer surface.

Jie et al. developed the first leaf-assembled TENG (Leaf-TENG), where the leaf cuticle from one side acted as a dielectric layer and the ion-rich mesophyll acted as an ionic conductor that can transfer its charge to an electrode connected to the external circuit.^170^ Poly(methyl methacrylate) (PMMA) was selected as the contact layer due to its significant difference in electron affinity in relation to the leaf surface, low-cost, high impact strength, and light weight. Under mechanical force, the leaf comes in contact with the PMMA sheet resulting in contact electrification at the interface. Since PMMA has a higher electron affinity than the leaf, negative charges will be transferred from leaf to PMMA. When PMMA is separated from the leaf, the changed electric potential difference will induce the movement of the ions in the interior of leaf, inducing a polarized electrical double layer at the electrolyte/metal interface which can transfer the electrons to the external circuit. Operated in single electrode mode under manual vibration in laboratory conditions, the Leaf-TENG provided a maximum power output of 45 mW m^–2^. The transient currents generated by the Leaf-TENG charged a capacitor that was used to power several LEDs and an electronic temperature sensor.

**Figure 20:**
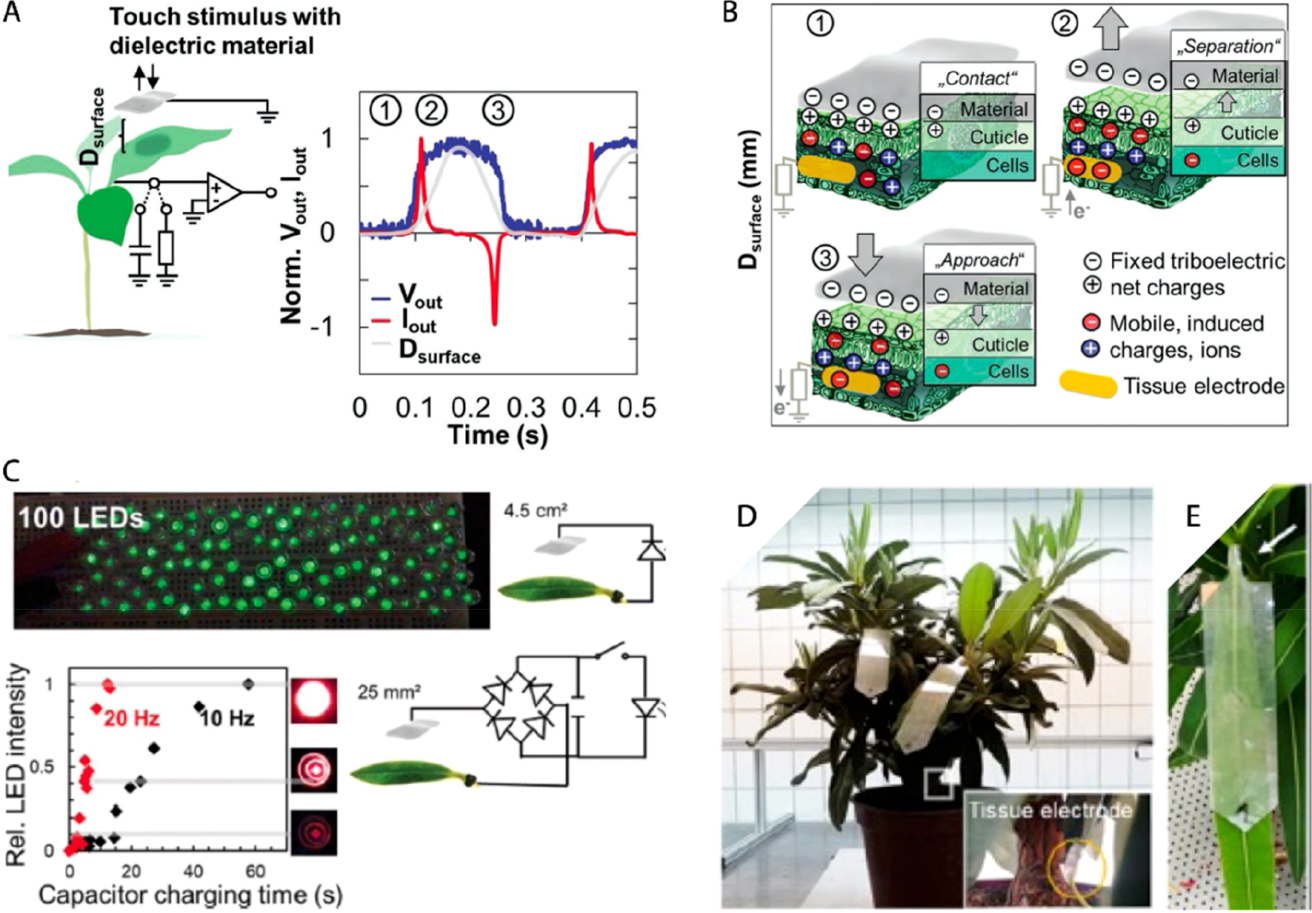
Triboelectric generators based on living plants for the harvesting of mechanical energy of wind. (A) Schematic diagram of the experimental setup and typical normalized output voltage and current generated when dielectric material touches leaf (measurement done with depicted circuit with capacitor or 100 MΩ load). (B) Working principle of the plant-based tribogenerator. (B) Mechanism of the tribogenerator working principle. (C) The developed tribogenerator is able to light 100 LED during each touch between a single *Rhododendron* leaf and Ecoflex pad. (D) Photography of living *N. oleander* with installed multiple artificial leaves for scaling up of the tribogenerator output. (E) Zoom in on single leaf with attached artificial leaf based on silicone rubber-coated indium tin oxide electrode on a PET support. (A–C) Reprinted with permission from ref (171). Copyright 2018 WILEY-VCH Verlag GmbH & Co. KGaA. (D,E) Reprinted with permission from ref (172). Copyright 2020 The Authors. Published by WILEY-VCH Verlag GmbH & Co. KGaA.

The first demonstration of triboelectric energy harvesting using a whole living plant and wind’s kinetic energy has been presented by Meder et al. (Figure 20A–C).^171^ By optimizing the dielectric material, the applied force, and the contact area, they reached power outputs up to 15 μW cm^–2^ under mechanical forces of 1 N in a single *Rhododendron yakushimanum* leaf with silicone elastomer dielectric. More recently, the same group also demonstrated the first study under outdoor-relevant conditions using *Rhododendron* and *Nerium oleander* for energy conversion upon various wind conditions and environmental humidity (Figure 20D,E).^172^ The generated voltage in TENGs gradually decreases with ambient humidity as it causes surface neutralization by the adsorption of counterions, but the performance recovers when RH is reduced.^173^ Several artificial silicone rubber-based leaves were coupled with multiple natural leaves that were connected using common electrodes in the plant stem. The generated energy was scalable with the number of leaves and wind speed depending also on the wind orientation. A maximum power of 300 nW was generated by eight leaves of *Rhododendron yakushimanum* and *N. oleander*, while using only four artificial modules enabled powering of 50 LEDs and a digital thermal sensor with display. Instead of attaching artificial leaves at the base of natural leaves petiole, Kim et al. fabricated a dielectric ribbon consisting of conductive fabric covered with silicone rubber. The ribbon was wrapped on a branch of *G. biloba* tree, forming so-called energy-harvesting vines.^174^ This approach yielded a maximum power of 3.97 W while operating four vines for 50 s enabled powering of three high-intensity LED bulbs for 2 min.

To further increase the environmental sustainability of power generators, Wu et al. presented fully biodegradable triboelectric generators using only the electrostatic charges that are present in nature.^169^ The generator converted the energy generated by the impact of water droplets on the leaf surface. In this case, one external electrode is placed on a leaf petiole while the other is located on the leaf surface. The leaf surface becomes charged with the impact of several droplets due to contact electrification. The droplets that hit the leaf also become charged forming electric double layer at the leaf/water interface. When the droplet spreads and touches the external electrode on the leaf surface, the circuit closes, and the charges are collected. Afterward, during the water droplet retraction, the electrons move backward to the plant tissue, yielding a current in a reverse direction. It was also shown that leaves with more hydrophobic surface were generating triboelectric energy more efficiently upon rain droplet impact. Because of its low affinity with water, the leaf would convert more efficiently the impact energy into electrostatic charges. Water impact on a detached *Mytilaria laosensis* leaf yielded a tens of mW m^–2^ power density range and an energy conversion efficiency of 0.2%.

### 7.3 Plants and Electricity

Every living organism requires mechanisms for signal transduction to coordinate their functions and respond to their environment. However, unlike animals, plants do not have a nervous system and therefore must rely on other more distributed signal transduction pathways for local and long-distance information processing and communication.

While there are still many gaps in the current knowledge about plant long-distance signaling pathways, there is consensus that these pathways can be broadly divided into three different categories: chemical signals, electrical signals, and hydraulic signals.^175^ It has also been suggested that the crosstalk between these pathways can be fine-tuned by transcription factors or turgor- and osmo-sensors,^176,177^ although the detailed understanding of this dynamic system remains elusive. Nonetheless, these long-distance signaling mechanisms have been shown to play an important role in various plant functions including responses to biotic and abiotic stressors, nastic movements and positive and negative tropism phenomena.

While hydraulic signals have been shown to regulate cellular turgor^178,179^ and appear as a key player in drought response,^177,180^ evidence that supports the theory of hydraulic signals as a long-distance signaling mechanism is still sparse in the current literature, and a critical interpretation of correlations should always be employed.^175^

Chemical signals include phytohormones, such as abscisic acid (ABA), jasmonate (JA), and salicylic acid (SA),^69,181,182^ ROS, volatile compounds, and ionic transients. For a detailed review on the different types of plant chemical signaling, the reader is referred to other works.^175,183^ While phytohormones are integrated in all aspects of plant growth and development and highly involved in biotic and abiotic stress response, their propagation speed, below mm s^–1^ range, hinders their potential as primary fast long-distance signaling molecules.^181,184^ However, there are indications of long-range transport via the vascular tissue,^185^ and regardless, these molecules remain highly involved in fast long-distance plant signal propagation, as will be explained below.

ROS, on the other hand, have been shown to propagate rapidly throughout plants^186^ and to interact with other signal transduction mechanisms, including electrical signals, calcium transients, phytohormones, and hydraulic signals.^187,188^ Other than ROS, calcium waves and electrical signals have been suggested as the main mediators of plant long-distance signaling.^189,190^ These latter signaling mechanisms are intrinsically related. Indeed, one important concept to highlight is the notion of electrical signal in the context of biological communication. In electrophysiology, electrical signals refer to signals that are of ionic origin and arise from ionic movements occurring within the organism, by the action of ion pumps, channels, and transporters, that lead to changes in membrane potentials. The collective movement of ions can be therefore recorded as a change in potential using electrodes that are placed intracellular, extracellularly or epidermal.^190^

While the first report on plant electrical signal dates back to the 19th century, when Burdon-Sanderson observed action potentials in the Venus flytrap,^191^ detailed knowledge about the biological nature of this communication and signaling remains in its infancy. As an example, while there is a consensus that plants respond electrically to artificial wounding, only few details are known about this response and the similarities/differences compared to natural wounding or other stimuli remain unexplored.^192^

What do we know now?

**Figure 21:**
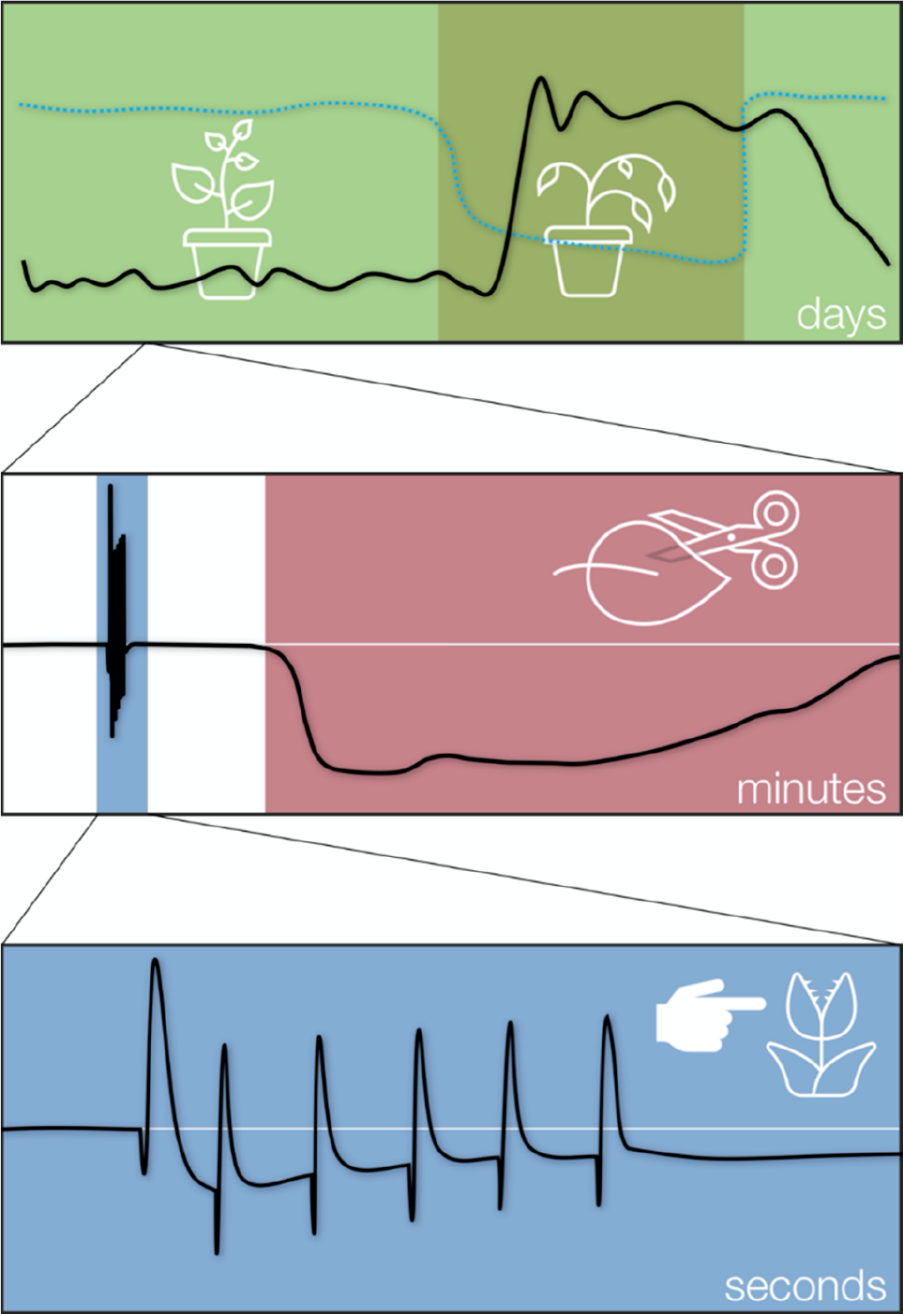
Different time scales of electrical responses in plants. While a drop in water availability (dashed blue line, top box) can elicit a change in surface potential that can last days, wounding or insect feeding leads to a slow-wave potential in the scale of minutes (red zone, middle box). Much faster than that, lasting only a couple of seconds, action potentials can be triggered by touching, in thigmonastic plants (blue zone in middle and bottom box).

The electrical signals in plants can broadly be divided in two types, related to their propagation speed. Fast signals are commonly described as action potentials, while slow signals are known as slow wave potentials or variation potentials (Figure 21). Action potentials refer to a thoroughly characterized electrical signal, widely described in animals. These signals are an “all-or-nothing” response, meaning that their amplitude and timing is only controlled by a threshold of membrane potential and spread to neighboring cells by serial depolarization.^193^ In plants, action potentials maintain the “all-or-nothing” nature due to the involvement of voltage-sensitive ion channels that induce a stereotypical response when a depolarization threshold is reached but are slower than those observed in animals,^194^ are propagated along the plasma membrane or tonoplasts, and are tightly related to calcium transients.^189^ Additionally, action potentials in plants are usually generated when mechanosensitive cells are triggered. The action potential then propagates and triggers changes in the turgor pressure of pulvinus cells that are in junction locations in the plant, and therefore, resulting in fast movement. The most typical examples of this type of signal are the *D. muscipula* (Venus flytrap, VFT) and *Mimosa pudica*.

In the case of the VFT, it had been hypothesized that mechanical stimulation of one hair would trigger a calcium transient in the neighboring sensor cells, which would propagate throughout the whole trap, with speed in the range of 0.2 m s^–1^.^195,196^ This calcium increase would not be enough to trigger the trap movement. Therefore, the closure would only occur if a second stimulation ensues within 30 s, raising cytosolic calcium concentrations above threshold and eliciting the closure.^197−199^ This hypothesis, widely accepted in the field, had only been indirectly demonstrated due to the lack of methods to study real-time ionic movement in plants. Indeed, when a VFT was incubated with calcium channel blockers, both the action potentials and consequent movement no longer occurred.^200^ The direct observation of calcium transients in VFT was only recently achieved. Suda and colleagues were able to observe calcium dynamics in real-time, using transgenic VFTs that express the calcium sensor GcaMP6f, which becomes fluorescent when in contact with cytosolic calcium. The authors were able to show that the calcium transient originates from the stimulated trigger hair and that it propagates anisotropically throughout the trap. Furthermore, the authors confirmed that a single touch cannot induce trap closure, but two touches within 30 s are required to increase cytosolic calcium concentration above the putative threshold.^201^ Importantly, while this new study sheds light on the involvement of calcium in plant movement, no cause-consequence relationship can be extracted, and further genetic and ionic studies are still required.

Slow wave potentials are more widespread among the plant population and are related to responses to external stimuli (wounding, heat, etc.).^202,203^ While these potentials also consist of transient changes in membrane potential, they differ from action potentials in their timing, being much slower with a delayed membrane repolarization, and in the variation of their amplitude, increasing in response with the stimulus intensity.^192,193^ Furthermore, while slow wave potentials can be transmitted over long distances, their amplitude decays with distance, are not able to self-propagate as efficiently as action potentials, and they are known to be induced by the inhibition of the H^+^-ATPase, which is responsible for the slower kinetics of these signals when compared to voltage-gated calcium channels.^193^

Importantly, a third type of plant electrical signal has been proposed: system potentials (SP). SP display apoplastic propagation and can spread throughout the organism. Differently from the other described signals, SPs are thought to be initiated by the activation of the H^+^-ATPase.^202^ It should however be noted that there is very little evidence regarding this type of signal, falling mostly in the theoretical field.

Even though both fast and slow electricals signals have been identified, many questions remain unanswered regarding their ionic origin, tissue of propagation and crosstalk with other signal transduction pathways such as calcium, ROS, and phytohormones. Regarding the involved tissues, while phloem vascular tissue has been suggested as the most probable conduit since it forms a connective ionic network throughout the plant, a recent study suggests that both xylem and phloem are involved in the propagation of electrical signals. To reach this conclusion, the authors explored the wound response in *Arabidopsis thaliana*, in which the glutamate receptor-like (GLR) channels are involved in membrane depolarization propagation. Using double reduced function mutants for these channels, the authors observed that the complete attenuation of electrical signal propagation only occurred in double mutants that affected both phloem- and xylem-specific GLRs, suggesting the simultaneous involvement of both vascular tissues in signal propagation.^204^

In an ionic perspective, these electrical signals have been shown to rely on calcium transients, originating from the apoplastic compartment,^205−208^ as well as on the spatial and temporally controlled production of reactive oxygen species.^189,209^ The complex relation between electrical signals, calcium transients, and ROS signaling is still not fully elucidated.^210^ However, recent studies point to an electrical-calcium coupling occurring in fast movement, as well as in wounding responses.^201,204^ Additionally, several observations also point to a coupling between calcium and ROS signaling in long-distance communication.^188^ The theory that calcium is in the basis of electrical transmission in plants has been further supported by the identification of voltage-gated calcium channels in the plasma membrane of plants that respond to changes in membrane potential by opening or closing and thus modulating ion fluxes.^194^ Furthermore, calcium dynamics in plants seem to display a higher complexity, since plant cells have been shown to access different sources of intracellularly stored calcium when exposed to different stimuli, which allows for different responses, both in magnitude and in timing, to different triggers.^211,212^ It should be noted that the minute relationship between electrical signaling and calcium transients remains ambiguous. While there is evidence that calcium is the driving force behind electrical signals,^201^ other studies show that the increase in cytosolic calcium only occurs after the maximum membrane depolarization is reached,^204^ suggesting the involvement of other molecules or messengers in the generation of electrical signals.

Finally, going full circle, recent evidence point to a crosstalk between electrical signaling and jasmonic acid signaling pathway. In *Arabidopsis*, wounding leads to the activation of GLRs, which modulate electrical activity and are in the basis of long-distance signaling. The correlation between wounding and increased JA-pathway gene expression was already observed in *Arabidopsis* systemic leaves.^213^ Importantly, since JA is known to counteract biotic stress and is involved in the response against herbivores, a key observation for the interaction between GLRs, electrical signals, and JA was recently accomplished, where larvae feeding on *Arabidopsis* GLR mutants gained more weight than those feeding on wild-type *Arabidopsis*,^204^ which suggests that the activation of the JA pathway is hindered in the absence of wound-induced electrical signal propagation.

In order to explore these versatile phenomena, several electrophysiological and optical techniques have been applied,^214^ although most studies in literature make use of single inorganic electrodes, with low spatial resolution. Among the most often encountered experimental setups, it is possible to find Ag/AgCl electrodes,^215,216^ stainless steel electrodes,^217−219^ Cu/Zn electrode pairs,^220^ KCl-filled glass microelectrodes connected to Ag/AgCl or Pt wires,^221,222^ and carbon steel bars,^223^ all generally used in pairs of one recording electrode and one reference, only providing electrophysiological information on one specific region of the measured plant. While very reliable and widely used, metal electrodes carry several disadvantages for plant electrophysiology, such as a significantly higher impedance when compared to conducting polymers, higher rigidity, and in some cases the need to be inserted in the plant tissue, which can elicit wound responses and consequent membrane depolarization, thus hindering the electrophysiological study of other phenomena. A different approach for the monitoring of plant electrophysiology consists of optical methods, which can employ voltage-sensitive dyes that reflect changes in electrical signaling in their own fluorescence, as illustrated by Bräuner and colleagues^224^ and, more recently, by Suda et al.,^201^ or can rely on ion-sensitive fluorescence probes, either genetically encoded^225^ or reversibly administered,^226^ that allow for real-time ionic mapping both in specific tissues and at the whole plant level.

However, some studies evade this norm, trying to minimize the invasiveness of the recording. One of such studies makes use of the “aphid method” technique,^227^ which relies on live aphids locating and feeding off the phloem. Following the attachment of its stylet to the phloematic vessel, the aphid is connected to a thin gold wire electrode, forming a stable and minimally invasive somewhat organic electrophysiology system.^192^ Other examples rely on electrochemical field-effect transistors (eFET) or microelectrode array systems to perform extracellular electrophysiological recordings, achieving a higher spatial resolution when compared to more traditional strategies.^214,228,229^

Looking into organic electronic devices, a recent study employed a conjugated-polymer-based organic electrochemical transistor (OECT), combining an active layer of poly[2,5-bis(3-tetradecylthiophene-2-yl)thieno[3,2-*b*]thiophene] and an ion exchange gel containing 1-butyl-3-methylimidazolium bis(trifluoromethylsulfonyl)imide and poly(vinylidene fluoride-*co*-hexafluoropropylene), where this device was able to outperform standard Ag/AgCl electrodes in the recording of action potentials in the VFT in terms of signal-to-noise ratio (100-fold difference).^230^ A different effort in organic electronic devices comprises the fabrication and characterization of printable PEDOT:PSS electrodes that can be used to perform surface measurements of electrical signals in different plants.^231^ In broad terms, the device is composed by PEDOT:PSS pads that are combined with a silver ink on a tattoo transfer paper. These electrodes represent an advancement in plant electrophysiology since they are conformable, being able to adapt to irregular plant surfaces; their adherence to plants is driven by van der Waals interactions, which suppresses the need for electrophysiology gel, or highly concentrated contact electrolytes; and their size (<3 μm in thickness) and lightweight are compatible with plant-based applications. These characteristics make them suitable for long-term measurements and allow them to be placed in mobile plant organs, such as *D. muscipula* traps or *C. motorius* moving leaflets.

## 8 Challenges and Outlook

Bioelectronics technologies for plants have only started to emerge even though the field of bioelectronics has been significantly advancing over the past decades for biomedicine. Studies for monitoring and modulating plant physiology are still at the proof-of-concept level, and in order for these technologies to show their true potential and to be established within plant science, more advanced studies have to be performed. Identifying the right biological questions where bioelectronics can be advantageous over conventional methodologies is an important task that can only be addressed by enhancing the collaboration between the bioelectronics and plant science communities. For implantable technologies, it is very important to understand the wound effect in plants various tissues and how it may or may not affect the readout. Initially, many of the technologies that have been developed for mammalian systems can be repurposed for use in plants while, as more research groups enter the field, we expect technologies to be specifically designed and developed for plant science.

**Figure 22:**
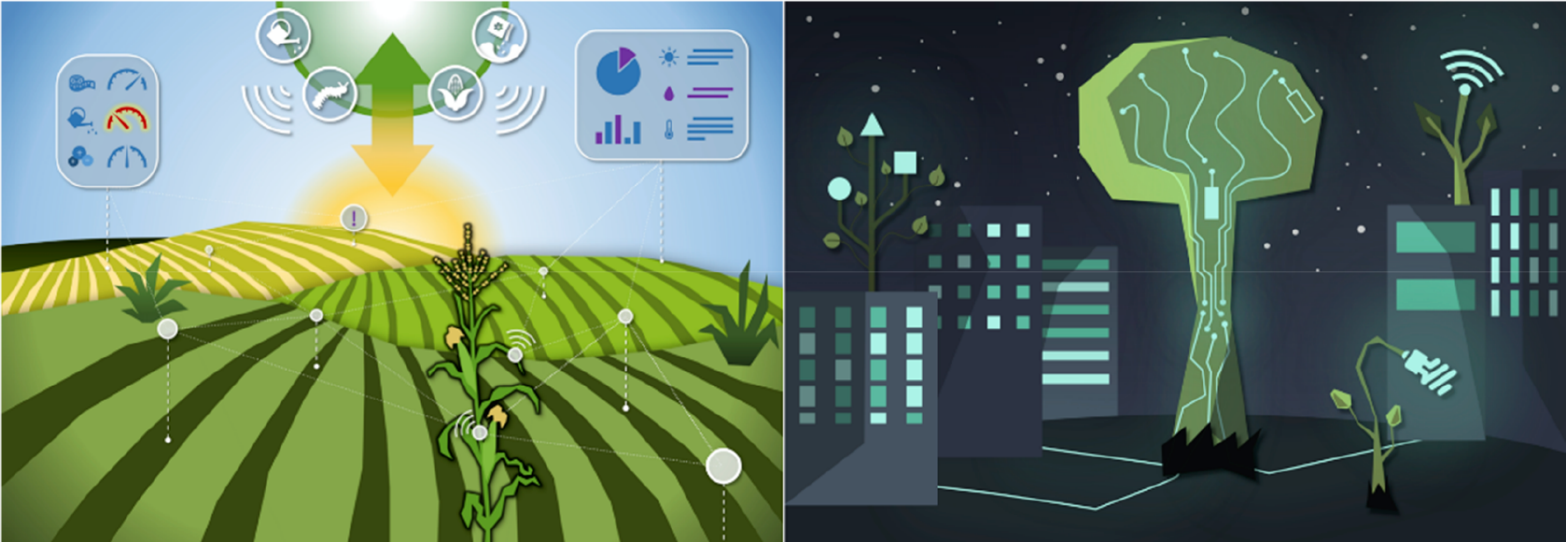
Future possibilities. On the left panel, fully integrated bioelectronic technology for smart agriculture enabling sensing, actuation, and decision making via distributed devices, cloud computing, and artificial intelligence. It is envisioned that the use of resources and yield are optimized. On the right panel, a futuristic urban environment with biohybrid plants, representing the potential for enhanced sustainable living. Street lighting, wi-fi antennas, and power sources are integrated in plants and seamlessly merged with city life.

For agriculture, a fully integrated technology that collects plant microclimate parameters in combination with plant physiological parameters and devices for regulating physiology will enable a whole new level of smart and precision agriculture. With advancements in big data analysis and AI-assisted decision making, one could envision a fully automated process from sowing to harvest (Figure 22). However, before reaching this level of sophistication, many challenges must be addressed. Large-scale integration of bioelectronic devices in thousands of plants will be costly and labor intensive, looking unrealistic. Then again, with the advancements on robotics, integration of devices can be automated. Otherwise, the technology can be integrated in selected plants that can be used as indicators for optimizing the growth of a larger number of plants. A grand challenge will be to translate the sensors information to practical action that can be implemented by the farmer. A lot of research has to be done to identify parameters of plant physiology that can be directly correlated with product quality and growth optimization. Furthermore, for field applications, one needs to consider fully integrated technology from powering to data collection and transmission. When discussing bioelectronic technologies for basic research in many cases high cost and complex protocols are not an issue, but this certainly will not be the case for translation of the technology into agriculture. Low-cost, large production with high fidelity, and simple use are prerequisites for successful commercialization of bioelectronic products. Another potential area of development is the agro-equivalent of point of care sensors for detecting diseases.

Finally, going from biomimetic systems to biohybrids can be a new technological revolution. From an exploratory research perspective, this is an exciting area of research that leads to a better understanding of how artificial materials and systems can be integrated into living organisms and form a bidirectional communication. In terms of applications, the studies in this case are, as well, at the proof-of-concept level with, for example, energy storage and harvesting and environmental monitoring that have so far not been integrated in real life settings. Further development is needed to increase performance, long-term operation, and identification of niche applications where biohybrid technologies can offer an advantage over conventional ones. Moreover, utilizing the biocatalytic cycles of plants for synthesis of materials that integrate into plant components could be employed for new generation of functional biopolymers, since concepts developed in plants have inspired applications in animals^232^ for in vivo synthesis of bioelectronic interfaces. On the other side of the coin, detailed investigation of plants’ movements offers immense possibilities for the development of novel soft robotic systems. For instance, a shape-shifting soft robot that grows in accordance with its environment, inspired by plant roots, has recently been realized.^233^ Furthermore, recent efforts on the detailed characterization of the mechanical properties of hook-climber stems aim to provide guidelines for the development of innovative robotic systems that can move and act in unknown environments.^234^

Plant-based biohybrid systems have already inspired the design and architectural community within the scope of “hybrid sustainable cities”. Our work on Electronic Plants inspired a speculative design project “Bionic Plants” where hybrid plants are envisioned as new species with integrated optimization and communication.^235^ Other projects have utilized plant electrical signals as readouts for controlling electronic devices.^236^ Particularly, Botanicus Interacticus uses a single electrode on the soil to map the plant’s reactions to touches, then assigns different touches to different functions, such as music creation and software control.^237^ Elowan is a “plant robot” that uses the plant’s electrical response to light to drive a wheeled robot toward the source of light.^238^ In the future, one might envision the biohybrid technologies discussed within this review fully integrated within the urban environment: light-emitting plants lighting our streets, trees as bulk batteries and power stations, and distributed plants as environmental sensors and communication towers (Figure 22).
